# Robust critical limb ischemia porcine model involving skeletal muscle necrosis

**DOI:** 10.1038/s41598-023-37724-7

**Published:** 2023-07-18

**Authors:** Mohamed S. El Masry, Surya C. Gnyawali, Chandan K. Sen

**Affiliations:** 1 McGowan Institute for Regenerative Medicine, Department of Surgery, School of Medicine, University of Pittsburgh, Pittsburgh, PA 15219 USA; 2 Indiana Center for Regenerative Medicine and Engineering, Indiana University Health Comprehensive Wound Center, Department of Surgery, Indiana University School of Medicine, Indianapolis, IN 46202 USA

**Keywords:** Biological models, Vascular diseases

## Abstract

This work sought to develop a robust and clinically relevant swine model of critical limb ischemia (CLI) involving the onset of ischemic muscle necrosis. CLI carries about 25–40% risk of major amputation with 20% annual mortality. Currently, there is no specific treatment that targets the ischemic myopathy characteristic of CLI. Current swine models of CLI, with tolerable side-effects, fail to achieve sustained ischemia followed by a necrotic myopathic endpoint. Such limitation in experimental model hinders development of effective interventions. CLI was induced unilaterally by ligation-excision of one inch of the common femoral artery (CFA) via infra-inguinal minimal incision in female Yorkshire pigs (n = 5). X-ray arteriography was done pre- and post-CFA transection to validate successful induction of severe ischemia. Weekly assessment of the sequalae of ischemia on limb perfusion, and degree of ischemic myopathy was conducted for 1 month using X-ray arteriography, laser speckle imaging, CTA angiography, femoral artery duplex, high resolution ultrasound and histopathological analysis. The non-invasive tissue analysis of the elastography images showed specific and characteristic pattern of increased muscle stiffness indicative of the fibrotic and necrotic outcome expected with associated total muscle ischemia. The prominent onset of skeletal muscle necrosis was evident upon direct inspection of the affected tissues. Ischemic myopathic changes associated with inflammatory infiltrates and deficient blood vessels were objectively validated. A translational model of severe hindlimb ischemia causing ischemic myopathy was successfully established adopting an approach that enables long-term survival studies in compliance with regulatory requirements pertaining to animal welfare.

## Introduction

Lower extremity peripheral arterial disease (PAD) is the stenosis of the aortoiliac segment to the pedal arteries either partially or completely^1^. PAD patients develop intermittent claudication, ischemic myopathy, and gait instability^2^. Critical limb ischemia (CLI) is the advanced stage of PAD, characterized by ischemic rest discomfort, non-healing ischemic leg and foot ulcers, and gangrene with both life and limb threatening complications^3^. Between 2000 and 2015, the global prevalence of PAD grew by 45%^4^. In the United States the prevalence of PAD at ≥ 40 years of age is estimated to be 7%^5^. CLI is a devastating disease that affects an estimated 0.5–1 million individuals each year. Up to 11% of PAD patients over the age of 50 developing CLI within five years of diagnosis^6^. CLI patients have high mortality, frequent amputations, or the persistence of CLI in the next year follow up^7^. Critical limb ischemia-related health-care expenses in Medicare patients have been projected to be approximately $5 billion per year^8^.

Beyond the medications provided for cardiovascular illness, PAD patients have few treatment choices, and none of them directly target ischemic myopathy^9^. Currently, surgical or endovascular revascularization is the primary intervention to treat CLI with few other therapies in routine clinical practice^1,9^. Additionally, there are several problems with the management strategies, including the challenge of selecting the appropriate and specific treatments for different patients. As a result, the need to test regenerative medicine solutions such as tissue nanotransfection based in vivo vasculogenic reprogramming, as reported by our group^10–12^, in large animal pre-clinical models is compelling. In the experimental study of ischemic pathologies, the distance between the point of vascular occlusion to the tissue at risk matters. In small animals, short distances may be confounded by ability of tissue oxygen to diffuse into the tissue at risk^13,14^. To approach human scale, the swine model of hind limb ischemia is commonly used^15–20^. Swine models of CLI currently reported in the literature successfully achieve transient ischemic insult^15,21–23^. However, necrotic myopathic endpoint, characteristic of CLI, is never achieved by a simple technique involving open surgical approach targeting one-vessel ligation with limited incision^15,16,21–23^. Lack of such simple experimental model is a critical barrier to test interventions for their ability to rescue ischemic myopathy. Thus, the objective of the current work was to develop a simple and robust clinically relevant swine model of CLI involving total and sustained ischemia causing the onset of skeletal muscle necrosis. As we sought to achieve that goal, we were mindful of the fact that to adequately test therapeutic agents while maintaining the animal welfare, the degree of ischemia must not be disabling or associated with severe morbidity.

## Results

### Ligation-Excision approach to induce hindlimb ischemia in swine with arteriography validation

**Figure 1:**
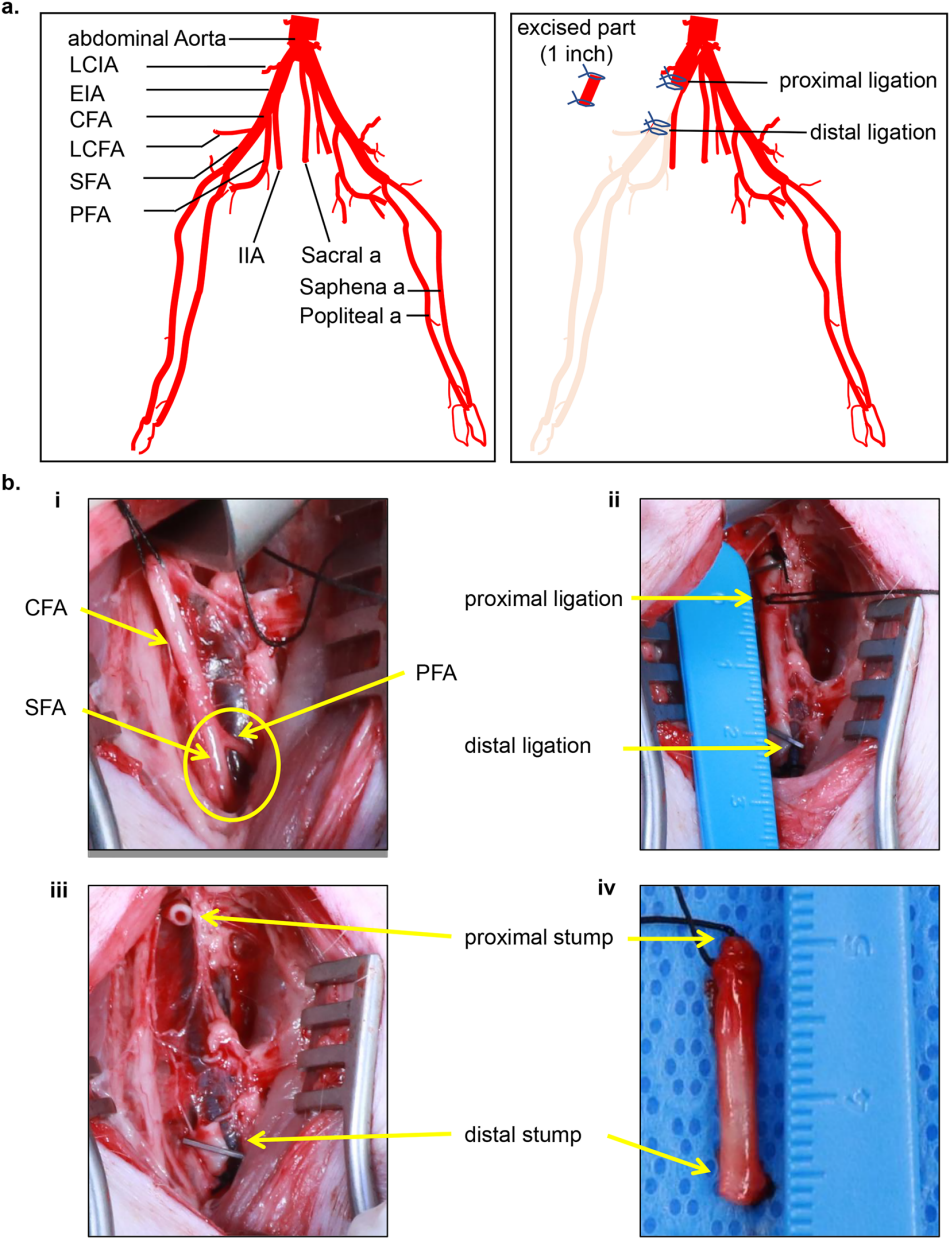
Ligation-Excision approach to induce hindlimb ischemia in Swine. (**a**) The reconstructed digitally subtracted serial fluoroscopy images of the pig hind limbs vascular tree on the right side with excision ligation of 1″ of the common femoral artery (CFA) and complete occlusion of blood flow to the hindlimb. (**b**) Infra-inguinal incision: i, CFA segment to be excised; PFA and SFA at the bifurcation are marked. ii, proximal and distal secured silk ligature of the CFA segment. iii–iv, excised CFA segment including secured silk ligature at the proximal and distal stumps of the artery. LCIA = lateral circumflex iliac artery; EIA = external iliac artery; IIA = internal iliac artery; CFA = common femoral artery; LCFA = lateral circumflex femoral Artery; SFA = superficial femoral artery; PFA = profunda femoris artery.

**Figure 2:**
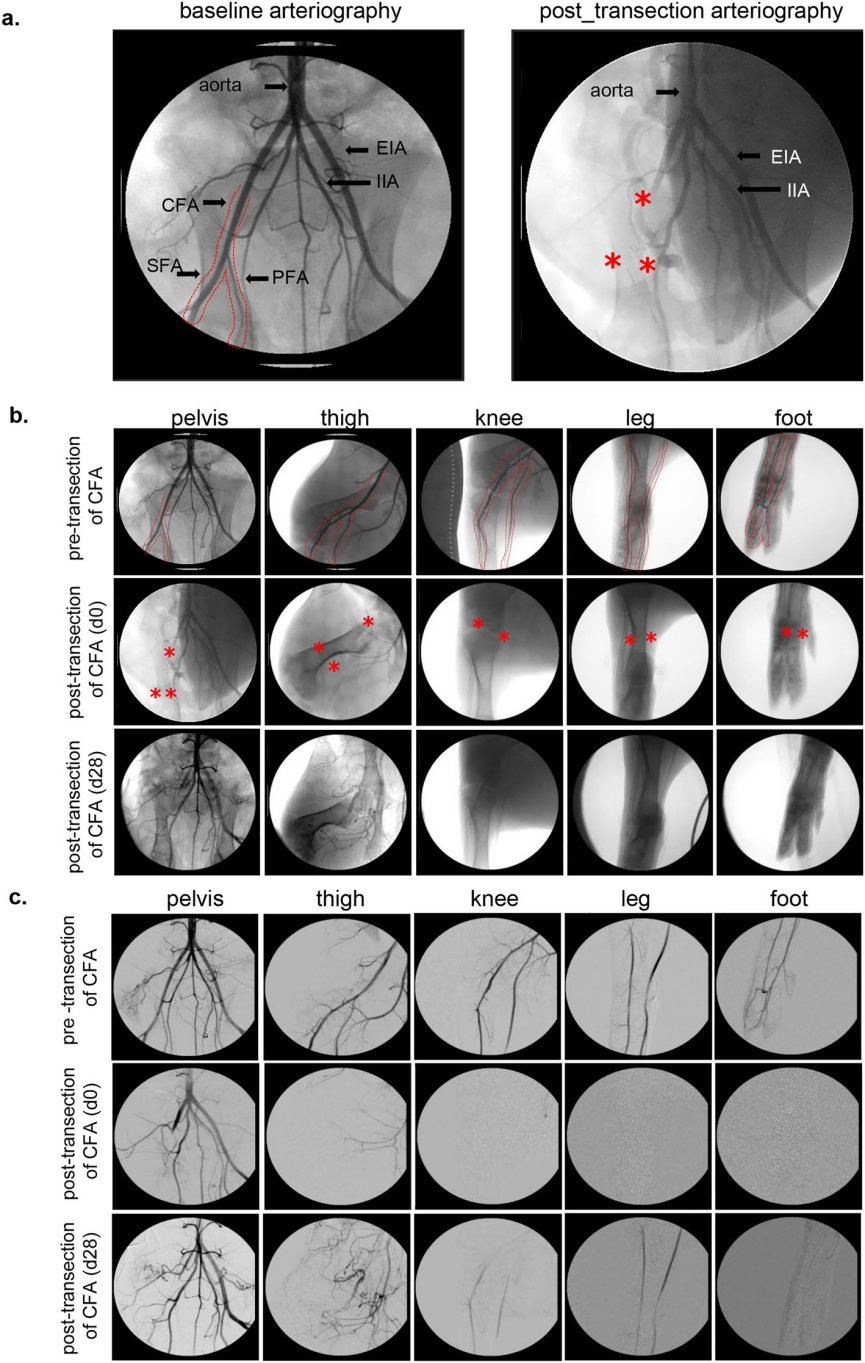
X-ray arteriographic characterization of hindlimb ischemia. (**a**) Representative arteriography images (left) visualizing baseline pig hindlimb arterial tree compared to the CFA post ligation excision (right). CFA, SFA and PFA are marked by red lines. EIA = external iliac artery; IIA = internal iliac artery; CFA = common femoral Artery; SFA = superficial femoral artery; PFA = profunda femoris artery. (**b**) Arteriographic images of different anatomical regions of the arterial tree demonstrating normal blood flow downstream of the planned (day 0) site of CFA excision. Excision of the CFA on day 0 was immediately followed by complete loss of blood flow. Development of collaterals didn’t impact the severity of the ischemia and ischemia was sustained until day 28 (day of sacrifice). CFA = common femoral artery. Asterisks denote arteries affected by excision. (**c**) Digitally subtracted arteriographic images over time (d0 pre-transection, d0 post-transection and d28 post-transection follow up) to demonstrate the arterial tree of the affected sites downstream of CFA.

**Figure 3:**
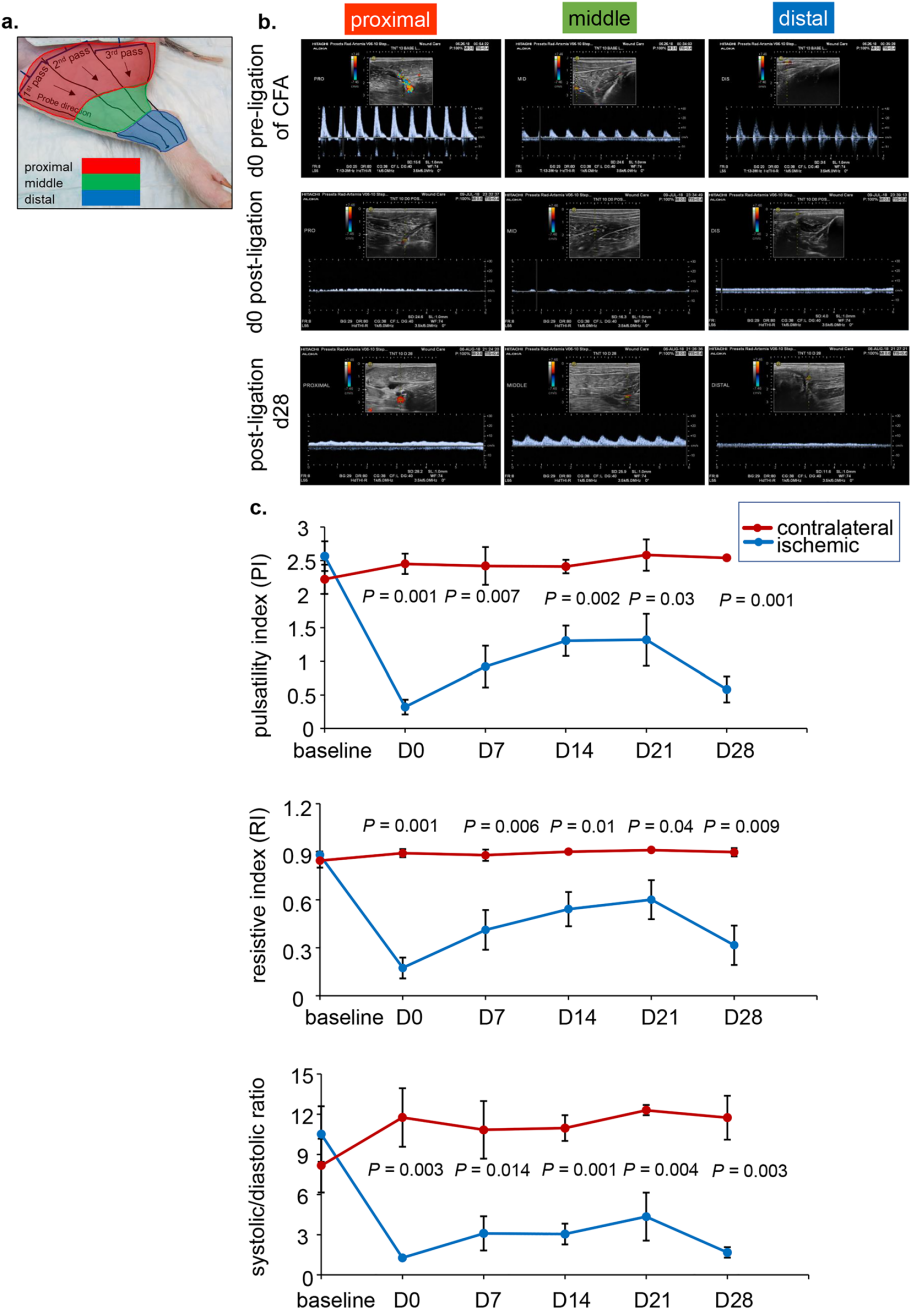
Validation of hindlimb ischemia by noninvasive Doppler ultrasound. (**a**) Anatomical regions of the hindlimb studied by Doppler ultrasound divided into proximal, middle, and distal along the thigh and leg. (**b**) Doppler images (still frame from video record) demonstrating normal Doppler waves at day 0 pre-ligation of the CFA. Following ligation-excision of the CFA, the waves are eliminated validating ischemia. Such ischemic condition was recorded until day 28 (sacrifice day). CFA = common femoral artery. (**c**) Doppler indices plotted show significant differences between the ischemic and contralateral limb. Differences in pulsatility index, resistive index and systolic to diastolic ratio are shown (n = 5). Data are represented as mean ± SD. Data were analyzed by two-tailed unpaired Student’s *t* test.

Surgical induction of ischemia was successfully achieved utilizing a clinically relevant preclinical porcine model (Figs. 1 and S1), by ligation-excision of 1 inch of the right CFA. The site of ligation was precisely localized using US and visual inspection of CFA following surgical dissection and confirmation by arteriography. Direct evidence of ischemia was intraoperatively recorded by real time arteriography (Figs. 2 and S2a,b). Furthermore, the ischemia was confirmed post-operatively by CTA (Fig. S2c) and doppler ultrasound imaging (Figs. 3 and S3). Localization of ischemia was achieved on day 0 following ligation-excision of the CFA by weekly x-ray arteriography which showed the complete occlusion of blood flow downstream of the site of vessel ligation before the bifurcation of the CFA in right hindlimb. Despite angiographic evidence demonstrating the limited development of collaterals, such partial reconstitution of distal vessel was insufficient to rescue the affected tissue against ischemic insult. Thus, we are led to infer that although the experimental ischemic insult caused some physiological adaptive responses, ischemic insult prevailed at a scale enough to sustain tissue damage until day 28 (day of sacrifice) (Figs. 2, 3). The region of ischemia extended from the level of CFA ligation to all the anatomical areas supplied by downstream blood vessels including thigh, leg, and foot (Fig. 2).

### Validation of hindlimb ischemia by noninvasive Doppler ultrasound

All Doppler indices of the CFA in the affected ischemic limb were significantly different, indicative of ischemia, compared to the contralateral hindlimb. Doppler color flow imaging and real-time velocity profiles are shown in Figs. 3a,b, S3. Measurement of the systolic/diastolic ratio of the ligated CFA was significantly lower than the ratio in the contralateral unaffected hindlimb at all timepoints assessed up to 4 weeks post-ligation. Furthermore, both PI and RI indices were significantly reduced compared to contralateral hindlimb and severely impacted by the ligation of the CFA. These changes persisted for 4 weeks triggering a cascade of permeant pathological changes in the blood vessels without sufficient collateral reflow (Fig. 3b).

### Impaired skin perfusion of the ischemic hindlimb

**Figure 4:**
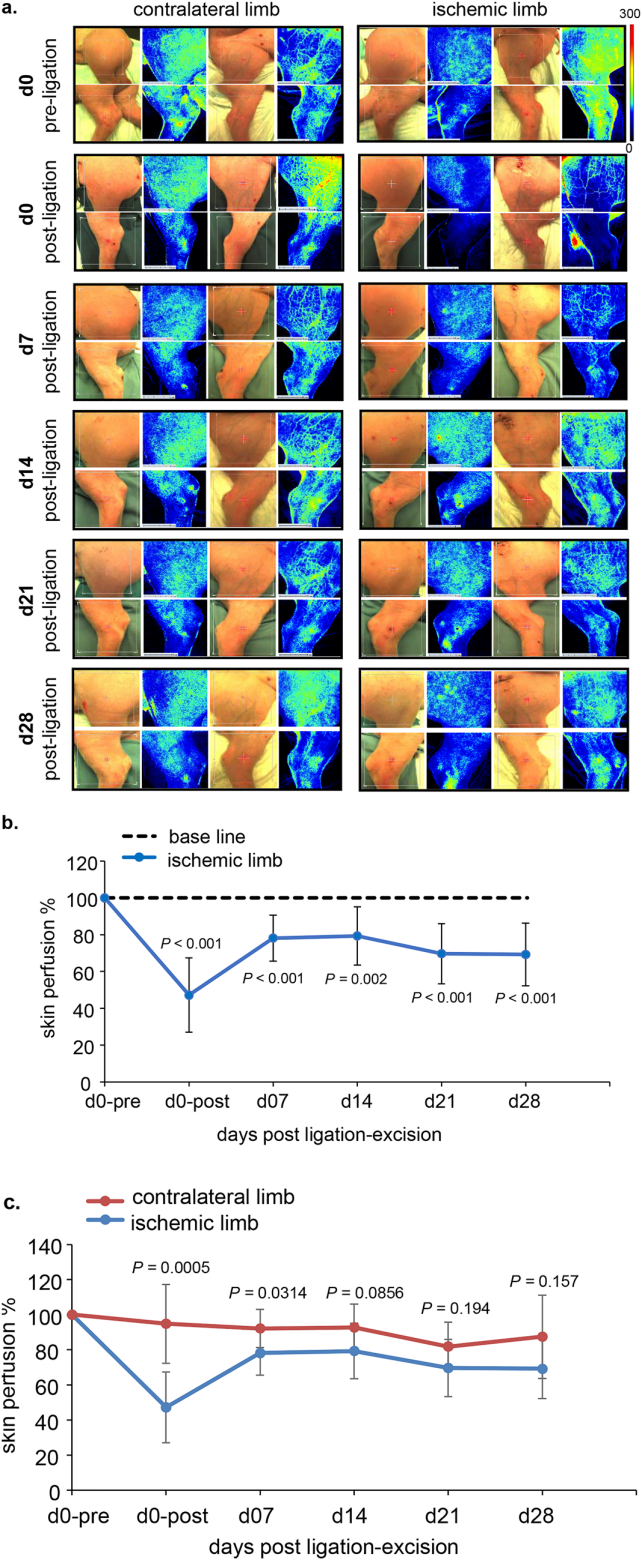
Hindlimb skin perfusion following surgical ischemia as determined by noninvasive laser speckle imaging (LSI). (**a**) Serial LSI images of hindlimb skin perfusion with the corresponding digital photos. Comparable skin perfusion is visualized in both contralateral hindlimb and the pre-ischemic right hindlimb at baseline (d0 pre-ligation). Hindlimb skin perfusion instantly diminished post ligation-excision of CFA. Such effect was sustained until d28 post-ligation. The sharp drop in skin perfusion following experimental ischemia was recorded by increased blue color relative to the green, yellow, and red colors in the LSI images. Scale bar = 5 cm. (**b**) Quantitative illustration of hindlimb skin perfusion of the ischemic hindlimb compared to the d0 pre-ligation (n = 5). (**c**) Quantitative illustration of hindlimb skin perfusion of the ischemic hindlimb compared to the contralateral limb (n = 5). Data are represented as mean ± SD. Data were analyzed by two-tailed unpaired Student’s *t* test.

To test the effect of surgically induced ischemia on hindlimb skin perfusion, LSI was performed weekly to determine changes in microcirculation (Fig. 4a–c). This experimental model is able to produce continuous sustained ischemia in all tissue components of the affected hindlimb. Serial assessment showed significant and profound deficit in skin perfusion in both inner and outer aspects of the thigh and leg starting immediately on day 0 post ligation-excision of the CFA. Such reduction of skin perfusion in ischemic limb reached 50% of the baseline and continued at levels of 70% up to day 28 (Fig. 4b). Normalization with reference to the contralateral limb showed significant deficit in skin perfusion on d0 post ligation up to day 7 post ligation, with slow and gradual restoration of skin perfusion at later time points (Fig. 4c).

### Myopathy in the ischemic hindlimb

**Figure 5:**
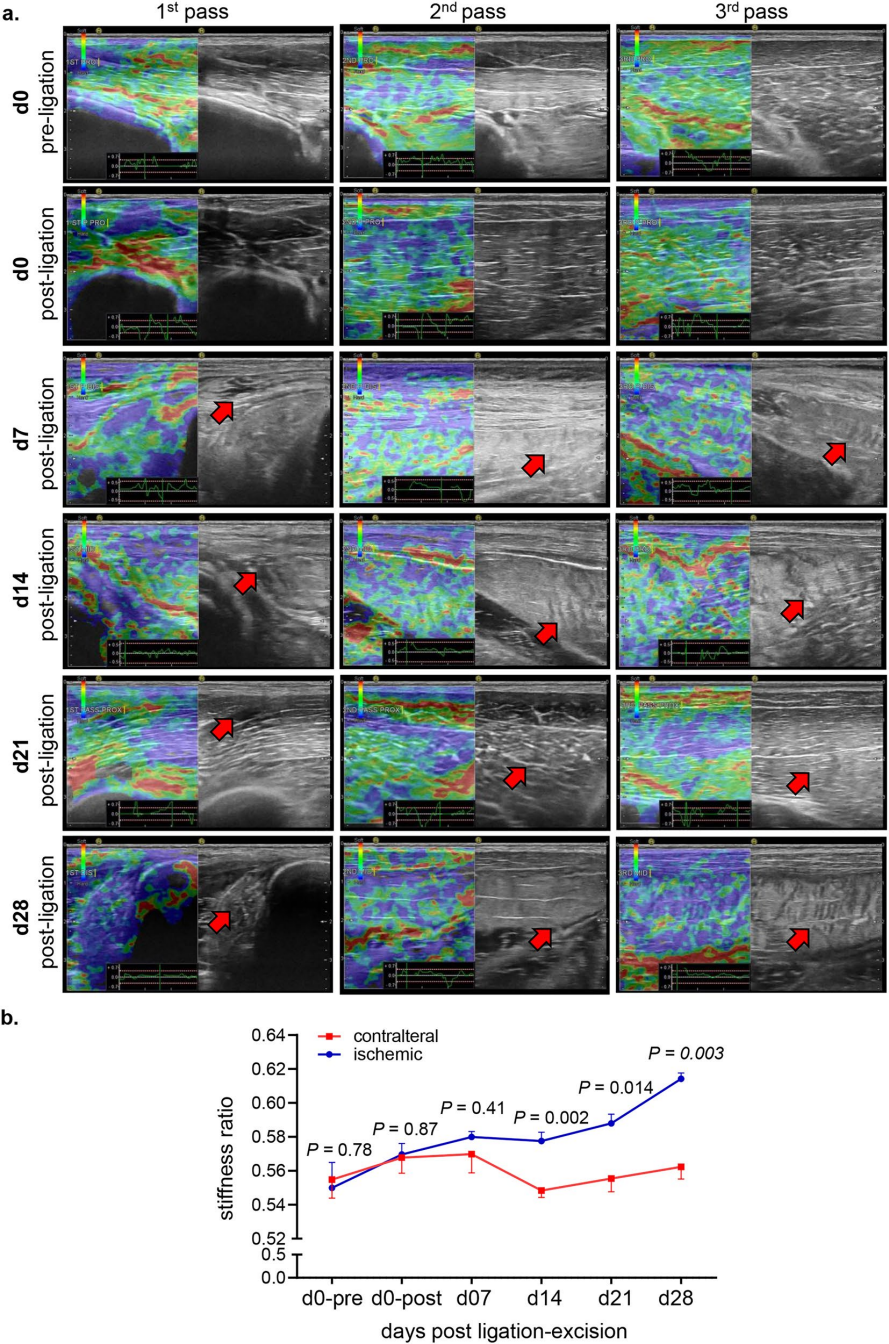
Structural (B-mode) and biomechanical (elastography) images of the hindlimb skeletal muscles in response to ischemia. (**a**) Co-registered (B-mode images, grey scale ultrasound and elastography, color ultrasound) ultrasound images characterizing baseline homogenous distribution pattern of skeletal muscle echo intensity (B-mode) and biomechanical strain (elastography) at d0 before induction of ischemia. Post-surgical weekly assessment illustrating the progression of ischemic myopathic changes in the form of increased muscle echo intensity (red arrows). The increase in muscle stiffness is represented by increase in blue colored regions over time until d28 (sacrifice day). For the ultrasound scans, the anatomical regions of the hindlimb were divided into proximal, middle, and distal along the thigh and leg. (**b**) Quantitative illustration of hindlimb muscle elastography in ischemic limb compared to contralateral limb (n = 5). Data are represented as mean ± SEM. Data were analyzed by two-tailed unpaired Student’s t test.

**Figure 6:**
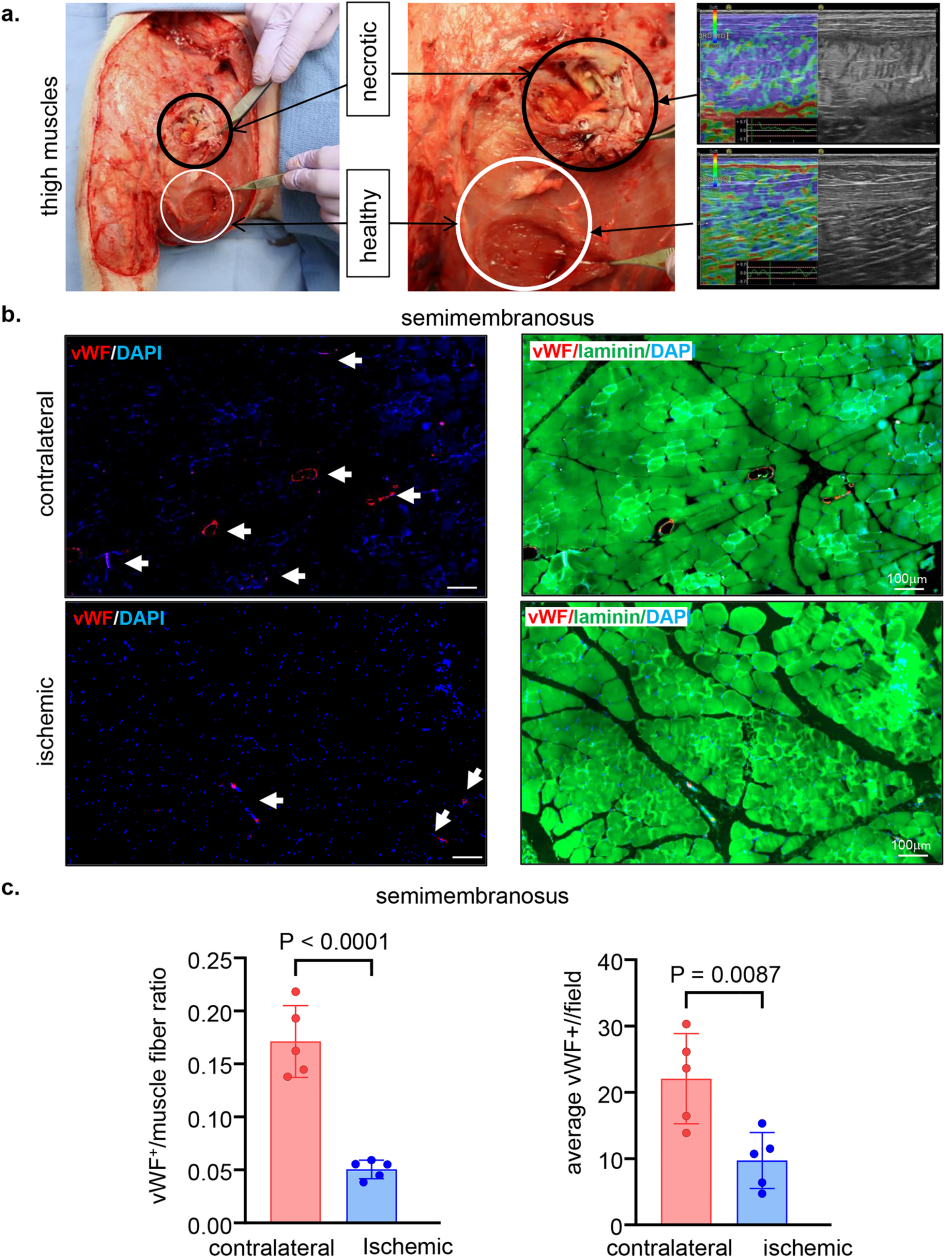

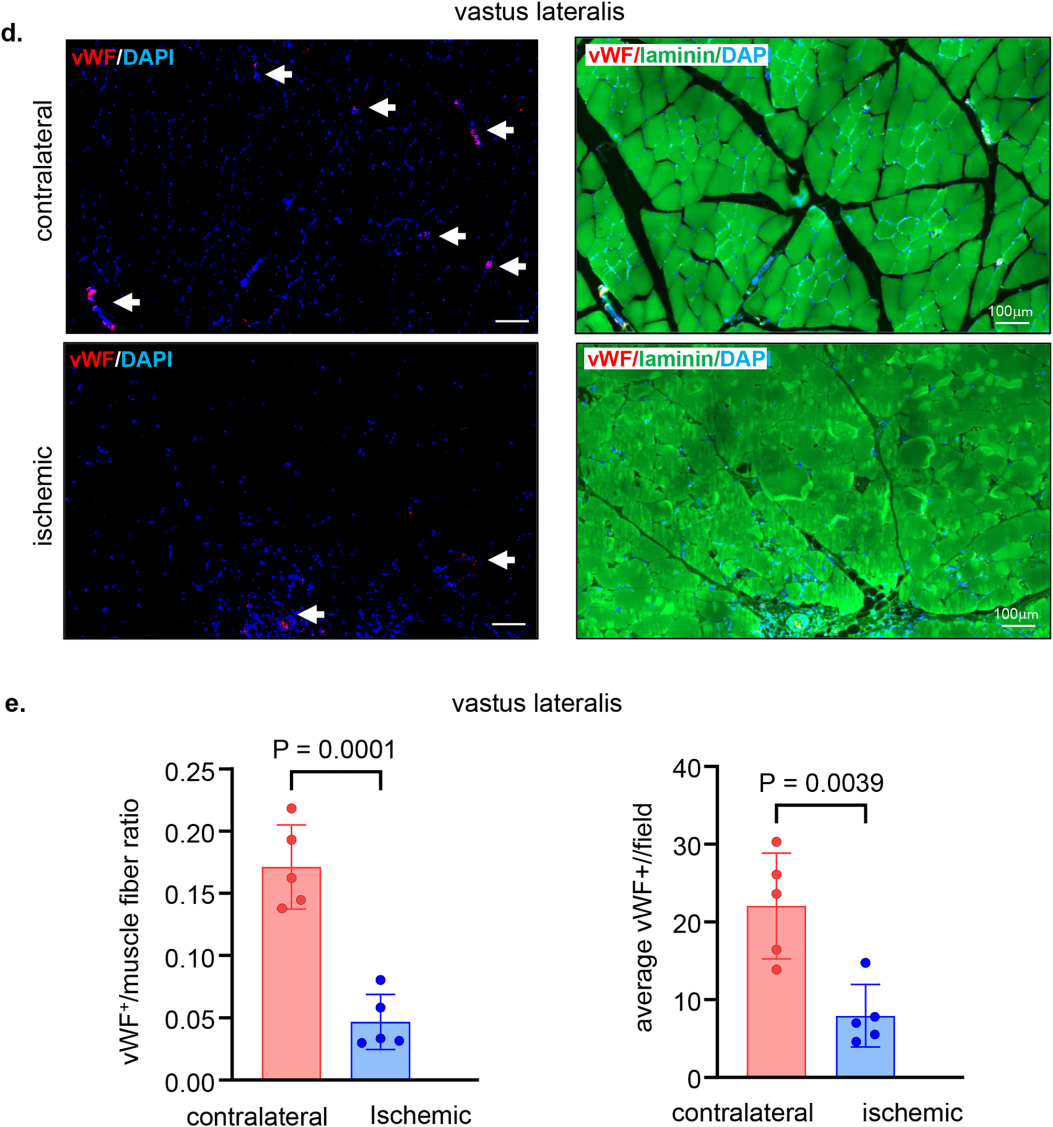

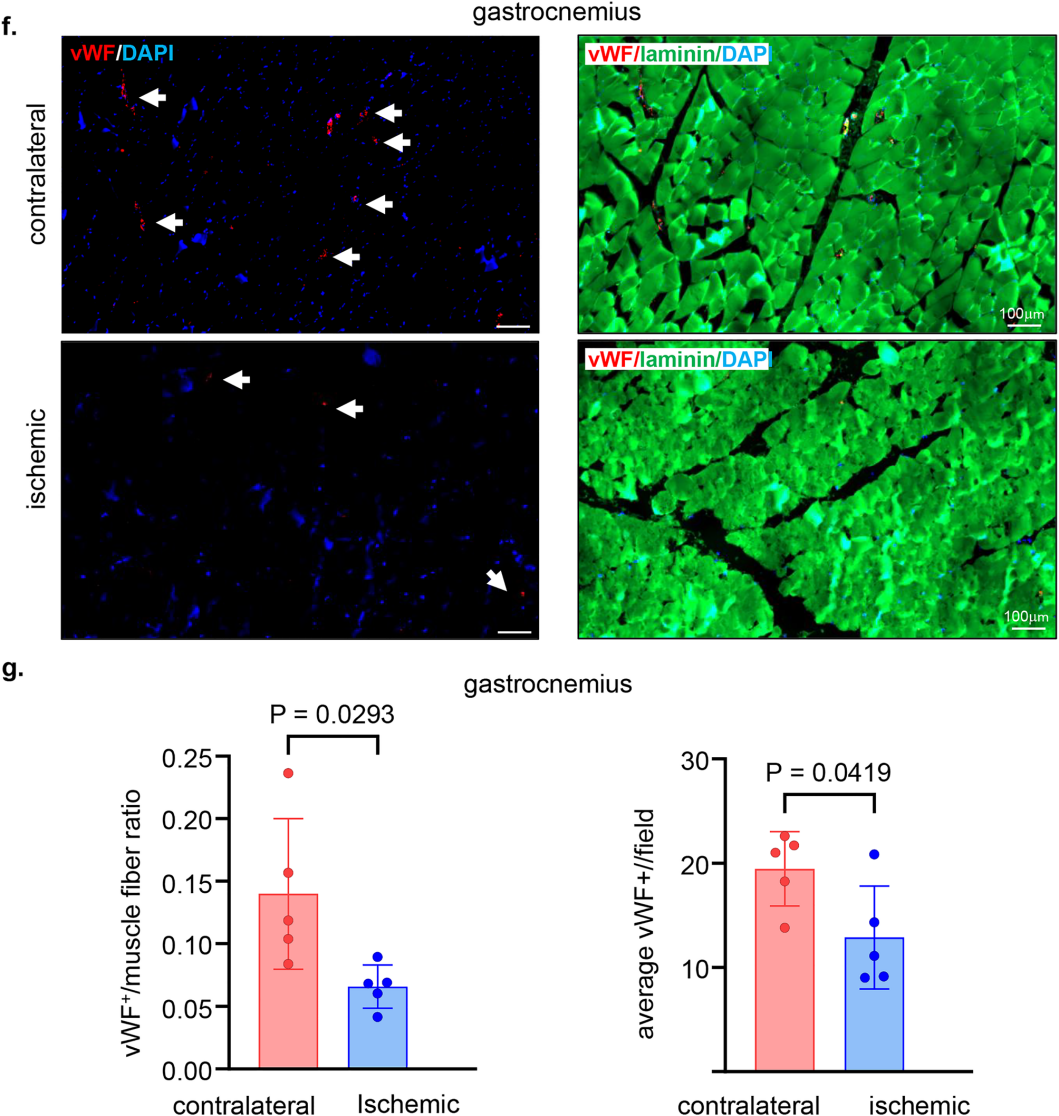

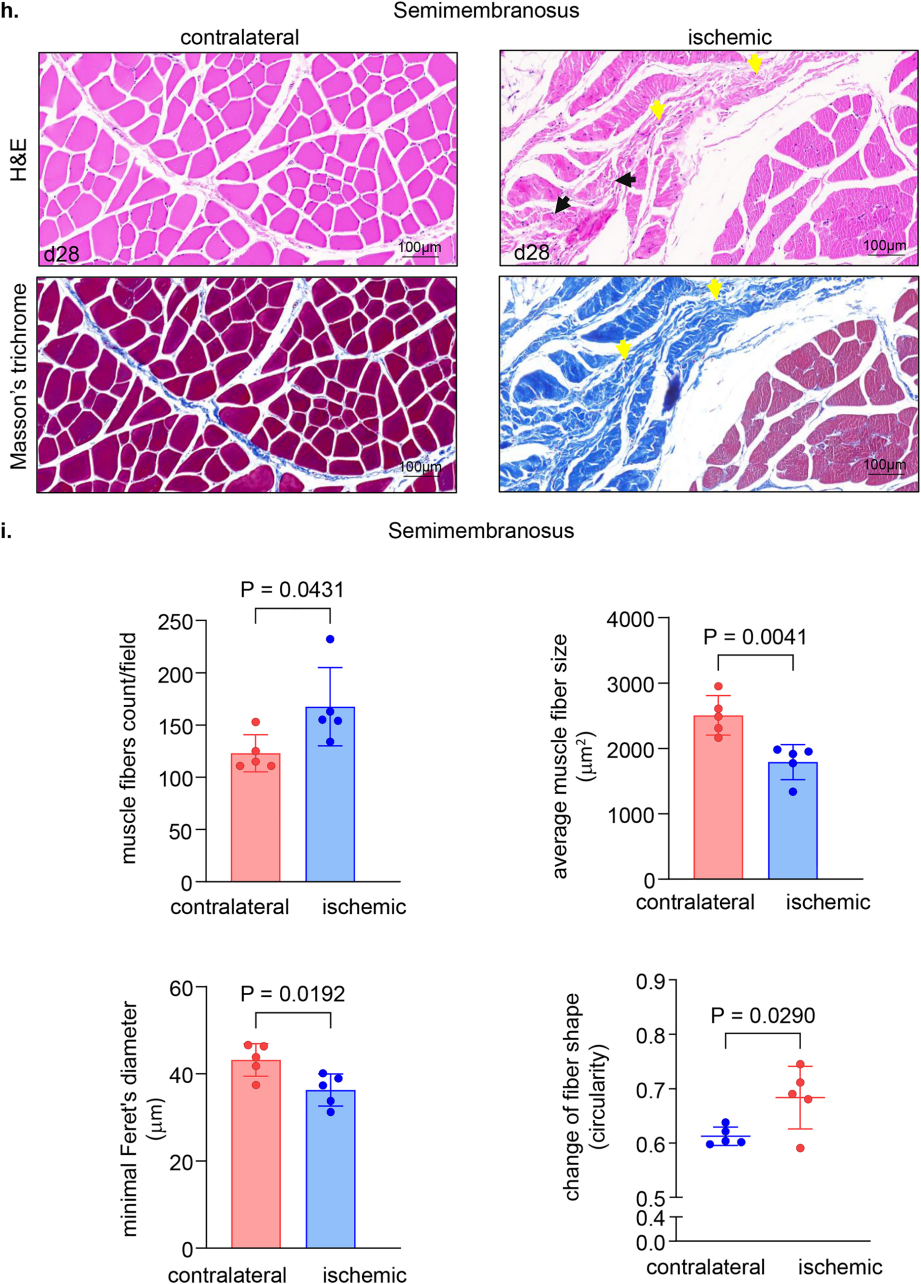

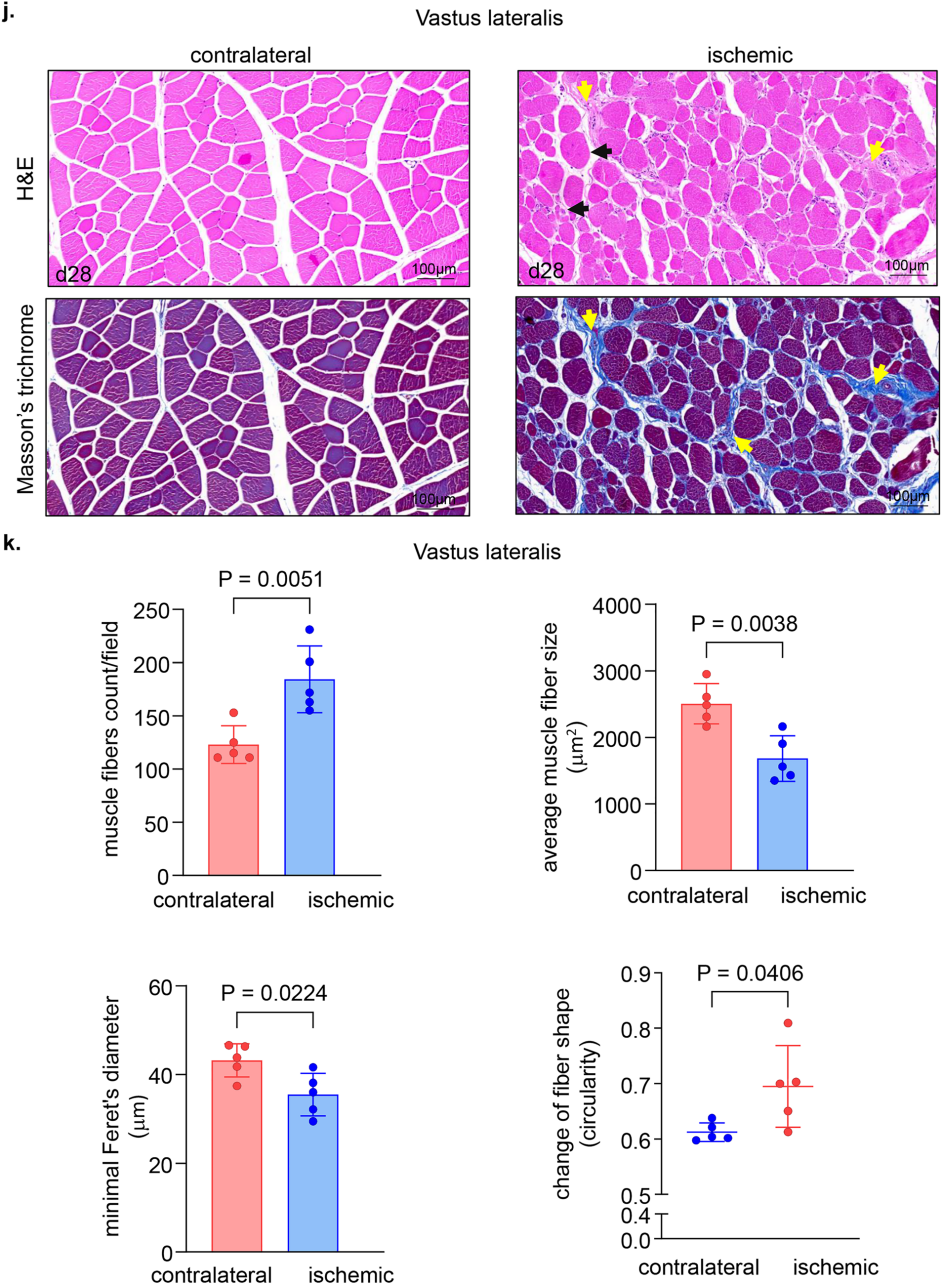

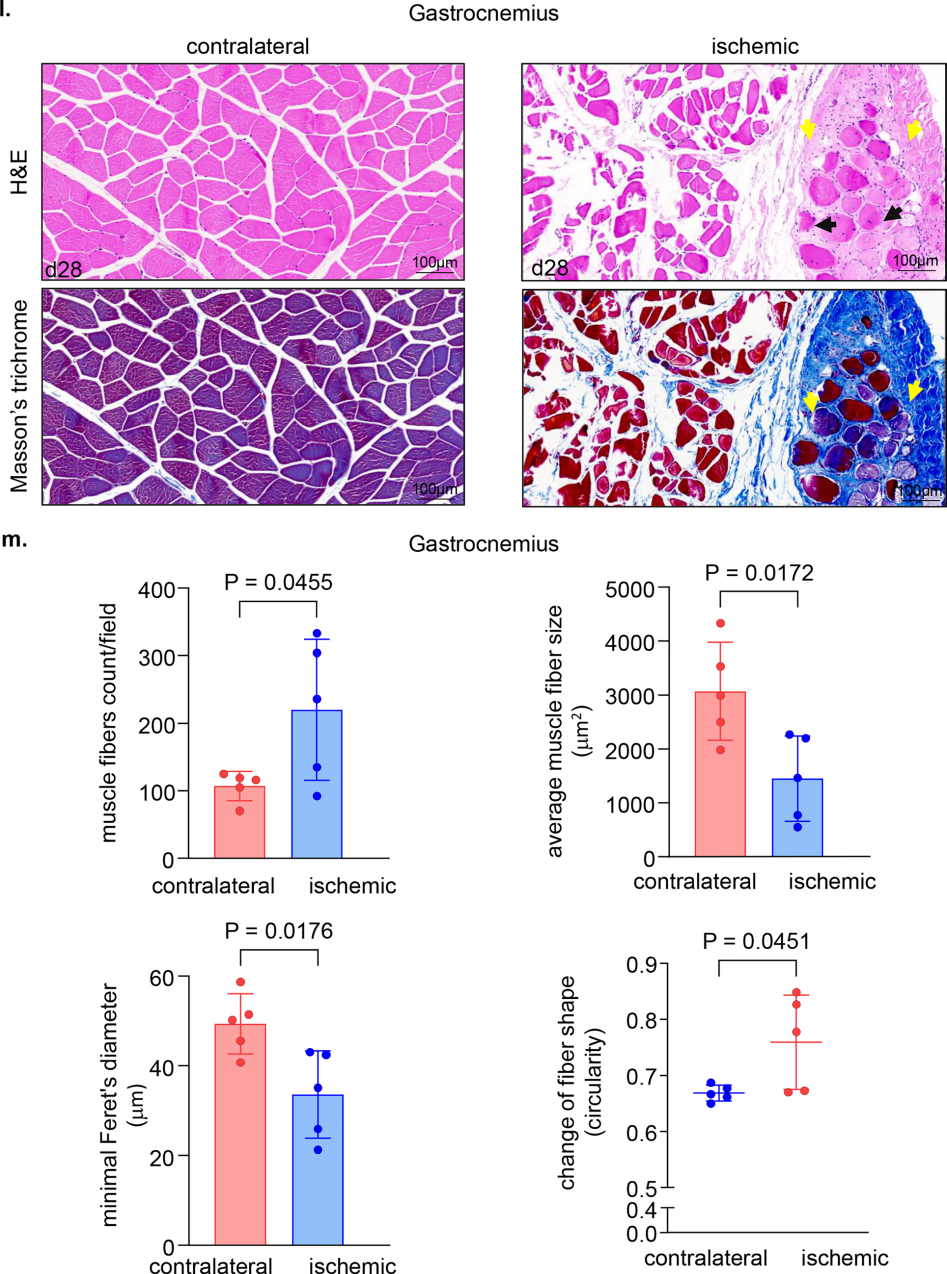
Macroscopic and microscopic validation of ischemic muscle necrosis. (**a**) Representative digital images demonstrating the d28 thigh muscle necrosis. The affected muscles are marked by black circle while healthy muscle are marked by white circle correlated with the corresponding changes (increase in blue colored regions) compared to healthy muscle in the elastography images. (**b**) Representative immunofluorescence staining of vWF (red) and laminin (green) with DAPI (blue) counterstaining in healthy contralateral muscles compared to the ischemic muscles (semimembranosus), white arrows point to identify blood vessels. Scale, 100 μm. (**c**) Quantification of blood vessels and blood vessels to muscle fibers per field for semimembranosus muscle. In the graph, each dot corresponds to the average of total of 10 ROIs from each muscle sample of both contralateral and ischemic muscles (n = 5). (**d**) Representative immunofluorescence staining of vWF (red) and laminin (green) with DAPI (blue) counterstaining in healthy contralateral muscles compared to the ischemic muscles (vastus lateralis), white arrows point to identify blood vessels. Scale, 100 μm. (**e**) Quantification of blood vessels and blood vessels to muscle fibers per field for the vastus lateralis muscle. In the graph, each dot corresponds to the average of total of 10 ROIs from each muscle sample of both contralateral and ischemic muscles (n = 5). (**f**) Representative immunofluorescence staining of vWF (red) and laminin (green) with DAPI (blue) counterstaining in healthy contralateral muscles compared to the ischemic muscles (gastrocnemius), white arrows point to identify blood vessels. Scale, 100 μm. (**g**) Quantification of blood vessels and blood vessels to muscle fibers per field for gastrocnemius muscle. In the graph, each dot corresponds to the average of total of ten ROIs from each sample of both contralateral and ischemic muscles (n = 5). (**h**) Representative cross-sectional H&E (upper panel) and Masson’s trichrome (lower panel) images showing histo-morphological changes in the ischemic muscles compared to healthy contralateral muscles (semimembranosus). Black arrows point to the abnormal and pathologic changes in affected myofibers which exhibited inflammatory cell infiltration and fibrosis as marked by yellow arrows. Scale, 100 μm. (**i**) Representative graphs of quantification of the muscle fibers count, morphological muscle changes, and Feret’s minimal diameter of semimembranosus muscle. In the graph, each dot corresponds to one muscle from each pig, which is the average of total of 7 ROIs from each muscle sample of both contralateral and ischemic muscles (n = 5). Data expressed as mean ± SD. Difference between means was analyzed by two-tailed unpaired Student’s *t* test. (**j**) Representative cross-sectional H&E (upper panel) and Masson’s trichrome (lower panel) images showing histo-morphological changes in the ischemic muscles compared to healthy contralateral muscles (vastus lateralis), Black arrows point to the abnormal and pathologic changes of the myofibers which associated with inflammatory cell infiltration and fibrosis marked by yellow arrows. Scale, 100 μm. (**k**) Representative graphs of quantification of the muscle fibers count, morphological muscle changes, and Feret’s minimal diameter of vastus lateralis muscle. In the graph, each dot corresponds to one muscle from each pig, which is the average of total of 7 ROIs from each muscle sample of both contralateral and ischemic muscles (n = 5). Data expressed as mean ± SD. Difference between means was analyzed by two-tailed unpaired Student’s *t* test. (**l**) Representative cross-sectional H&E (upper panel) and Masson’s trichrome (lower panel) images showing histo-morphological changes in the ischemic muscles compared to healthy contralateral muscles (gastrocnemius), Black arrows point to the abnormal and pathologic changes of the myofibers which associated with inflammatory cell infiltration and fibrosis marked by yellow arrows. Scale, 100 μm. (**m**) Representative graphs of quantification of the muscle fibers count, morphological muscle changes, and Feret’s minimal diameter of gastrocnemius muscle. In the graph, each dot corresponds to one muscle from each pig, which is the average of total of seven ROIs from each muscle sample of both contralateral and ischemic muscles (n = 5). Data expressed as mean ± SD. Difference between means was analyzed by two-tailed unpaired Student’s *t* test.

The non-invasive tissue analysis by elastography showed specific and characteristic pattern of increased muscle stiffness indicative of the fibrotic and necrotic outcome expected with total muscle ischemia (Figs. 5a, b and S4). These color-coded images, extracted from the elastography recorded videos by US, depicted significant increase in the blue regions (stiff) compared to red and yellow regions^24,25^. This pathological stiffness of the muscles was prominent by day 14 after ischemia and steadily increased over time until day 28 (Fig. 5b). Direct characterization of the hindlimb muscle pathology was based on muscle biopsies collected 4 weeks after the onset of ischemia. The prominent presence of skeletal muscle necrosis was evident upon direct inspection of the affected tissues (Figs. 6a, S5). Histopathological analyses revealed deficient vascular tissue accompanied with heavy inflammatory cell infiltration and myofiber pathology (Fig. 6b–m). The density of the vascular tissues to the muscle fibers ratio was significantly lower in the ischemic muscle compared to the contralateral healthy muscles (Fig. 6b–g). Ischemic muscle fibers showed significant micromorphological changes demonstrated by centrally located nuclei, loss of interstitial space with heavy inflammatory cellular infiltrate compared to the regular polygonal shape, peripherally located nuclei, with preserved interstitial space and no inflammatory infiltrates in the healthy muscle fibers (Fig. 6h–m). Objectively, the muscle fiber count, size, minimal Feret’s diameter and the change of the fiber shape were significantly lower in the ischemic limb compared to the contralateral limb in all different muscles studied including semimembranosus (Fig. 6h,i) and vastus lateralis (Fig. 6j,k) from the thigh region and gastrocnemius muscle (Fig. 6l,m) from the calf region. These changes represent the pathological and degenerative effects of long-term ischemia in the affected hindlimb.

### Neuropathy in the ischemic hindlimb

**Figure 7:**
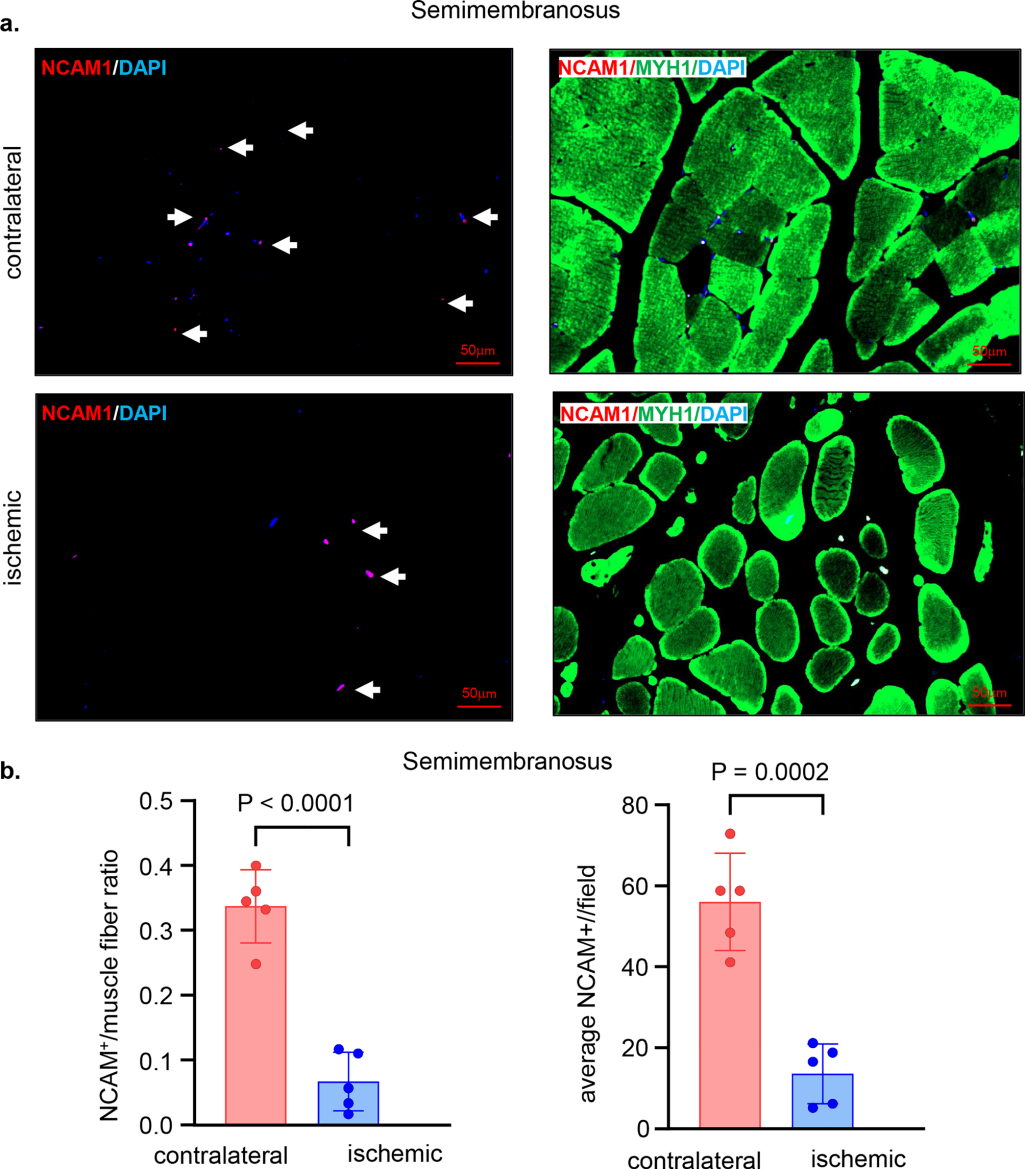

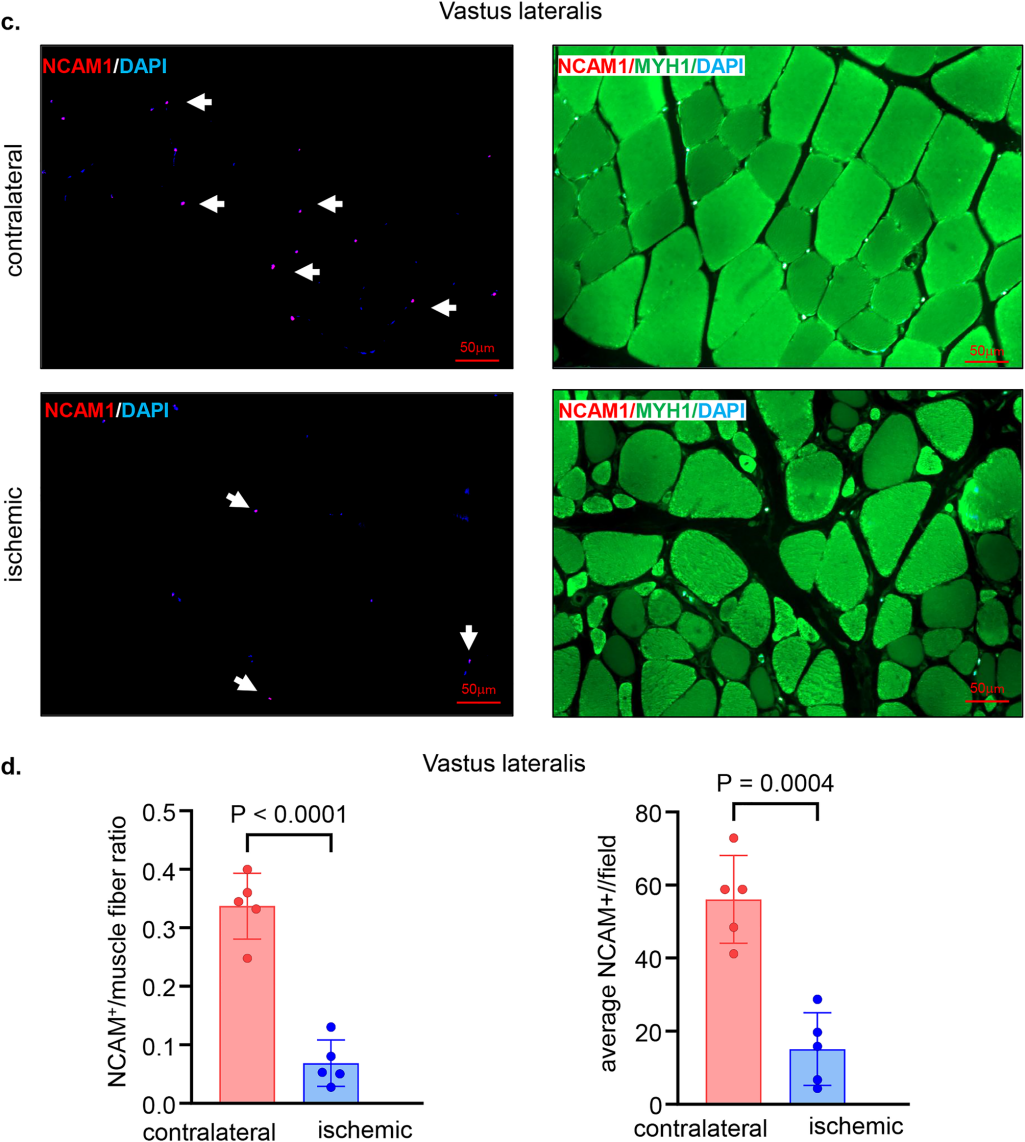

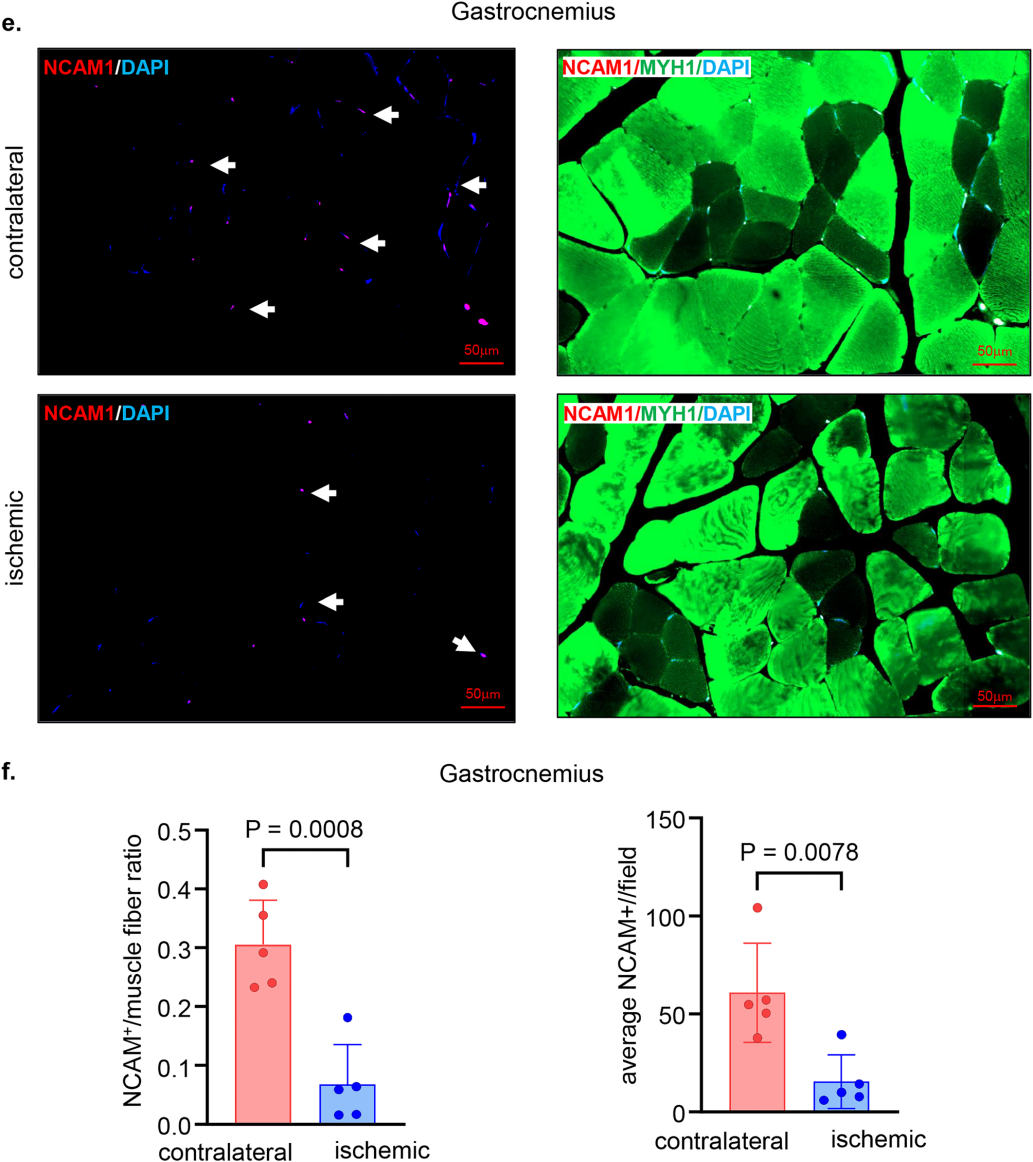
Low skeletal muscles NCAM1 mark ischemic neuropathy. (**a**) Representative immunofluorescence staining of NCAM1 (red) and MYH1 (green) with DAPI (blue) counterstaining in healthy contralateral muscles compared to the ischemic muscles (semimembranosus), white arrows point to the NCAM1^+^ muscle fibers. Scale, 50 μm. (**b**) Quantification of NCAM1^+^ and NCAM1 to muscle fibers per field for semimembranosus muscle. In the graph, each dot corresponds to the average of total of 7 ROIs from each muscle sample of both contralateral and ischemic muscles (n = 5). (**c**) Representative immunofluorescence staining of NCAM1 (red) and MYH1 (green) with DAPI (blue) counterstaining in healthy contralateral muscles compared to the ischemic muscles (vastus lateralis), white arrows point to the NCAM1^+^ muscle fibers. Scale, 50 μm. (**d**) Quantification of NCAM1^+^ and NCAM1 to muscle fibers per field for vastus lateralis muscle. In the graph, each dot corresponds to the average of total of 7 ROIs from each muscle sample of both contralateral and ischemic muscles (n = 5). (**e**) Representative immunofluorescence staining of NCAM1 (red) and MYH1 (green) with DAPI (blue) counterstaining in healthy contralateral muscles compared to the ischemic muscles (gastrocnemius), white arrows point to the NCAM1^+^ muscle fibers. Scale, 50 μm. (**f**) Quantification of NCAM1^+^ and NCAM1 to muscle fibers per field for gastrocnemius muscle. In the graph, each dot corresponds to the average of total of 7 ROIs from each muscle sample of both contralateral and ischemic muscles (n = 5). Data expressed as mean ± SD. Difference between means was analyzed by two-tailed unpaired Student’s *t* test.

Histopathological analyses of the ischemic skeletal muscles revealed deficiencies in the neuromuscular junctions as evident by the significantly lower numbers of NCAM1 positive muscle fibers in the ischemic muscles compared to that in corresponding contralateral muscles (Fig. 7a–f). The ischemic neuropathy affected different muscles including semimembranosus (Fig. 7a–b) and vastus lateralis (Fig. 7c–d) from the thigh region and gastrocnemius muscle (Fig. 7e–f) from the calf region.

## Discussion

Over the years, progress in the clinical care of CLI patients has been limited such that PAD and diabetes mellitus are responsible for 90% of major lower extremity amputations in the United States and Western European countries^26^. CLI, including both life and limb threatening complications, represents a severe and advanced stage of PAD. Ischemic myopathy of the lower extremity and related muscle damage represents a hallmark of CLI reflecting extreme severity of the ischemia^27^. PAD, in its clinically presented form, includes signs of skeletal muscle pathology secondary to ischemia. Such ischemic myopathy is known to be debilitating for CLI patients^27,28^. Surgical revascularization, aimed at reperfusion^29^, is classically reserved for late-stage patients who didn’t respond to medical therapy or who have advanced to CLI. At that point of disease progression wherein ischemic myopathy has set in, surgical revascularization may not always be sufficient to prevent amputation^8^. This work fills the unmet need of a large animal CLI model with clinically relevant disease severity including ischemic myopathy of the affected extremity.

The history of medicine assigns a powerful role to appropriate pre-clinical disease models in developing novel therapies^30^. In ischemic disorders, graded extent of damage to the tissue based on its distance from the site of occlusion is commonly evident^27,31^. In the setting of CLI, such distances span across the entire limb involving a scale that cannot be addressed in small animals^13,14^. Thus, a large animal model of severe hindlimb ischemia causing ischemic myopathy must be established adopting an approach that enables long-term survival studies in compliance with regulatory requirements pertaining to animal welfare. At present, several models of hindlimb ischemia in swine are reported^15–20^. These models provide several critical advantages, functional and hemodynamic, with respect to human translational value^32^. Furthermore, clinical imaging modalities are directly applicable to swine hindlimb ischemia models^33^. With the exception as discussed below, all such models, involving approaches that would allow long-term survival studies, have failed to achieve hind-limb ischemia of a severity that would cause ischemic necrosis of the affected limb muscles^15,21–23^. In one study reporting necrotic muscle damage of the affected hindlimb, occlusion was achieved by aggressive triple ligation of ipsilateral external iliac and bilateral internal iliac arteries^16^. A major limitation of occluding the bilateral internal iliac arteries is the unwanted involvement of pelvic ischemia which will cause side-effects^34,35^. Adverse side effects, expected under conditions of laparotomy and pelvic ischemia, such as hernia and infection were reported^16^. Large incisions, extending from mid abdomen to thigh, as employed in the above study are undesirable in survival surgery models^20^. Ischemic muscle damage was reported by endovascular approach^17^. Endovascular approaches require specific setup and skills, and with known related complications^36–39^.

The approach presented in this work achieves CLI-relevant necrotic extremity muscle damage while leaving the pelvic circulation unaffected. Additionally, the minimal incision open ligation-excision approach is procedurally simple and does not require endovascular skills. Other studies not involving the internal iliac arteries, achieved hindlimb ischemia of a lower severity not including necrotic damage of the affected skeletal muscle^15,21–23^. In other related approaches involving occlusion of the superficial femoral artery^15,23^ only partial ischemia was achieved because of active profunda femoris artery (PFA) circulation. In addition, such procedure induced collateral anastomosis between the open profunda and ligated superficial femoral artery^15,20^. These outcomes added to the residual circulation of the affected hindlimb downgrading the insult to partial ischemia incapable of causing ischemic myopathy.

During this work, preparatory studies involving the occlusion of the superficial femoral artery caused partial ischemia as validated by X-ray arteriography. Such finding is consistent with observations reported in independent studies^15,21,23^. Total ischemia was not achieved because of shunting of blood flow to the deeper vessels such as PFA. Limited inguinal incision with CFA ligation-excision was effective in achieving total ischemia. Such procedure did not result in collateral development that would comprise the severity of ischemia. Although we didn’t calculate Ankle Brachial Index (ABI), we have used different doppler parameters to assess tissue perfusion and vessel conditions that are objective and of research grade. When human patients are clinically presented, the time point is far downstream from the onset of ischemia. At such late stage, ulceration and gangrene are expected. In the model we present, we didn’t observe skin gangrene because we are few weeks away from the onset of ischemia where necrotic muscle damage appears as the first pathological manifestation. Successful early detection of the onset of ischemia and appropriate upstream intervention to rescue will spare such ulceration and gangrene. This model is developed to test such interventions. Our ligation excision approach of a healthy vessel does not represent the slow occlusive process of atherosclerosis and the associated pathological changes. However, the current approach isolates the blood supply variable of PAD and allows for specific study of that factor.

Robustness of the reported CLI model is validated by a comprehensive approach employing both invasive as well as non-invasive techniques supported by quantitative analyses. Following CLI surgery, not only the perfusion of the affected muscle but that of the skin remained compromised over time indicating a comprehensive effect on the hindlimb as is typically noted in humans^40,41^. It is recognized that on day 28 following ischemia, angiography showed some appearance of reconstitution of the distal vasculature perhaps as an adaptive response. Importantly, such response was unable to influence the severity of ischemia. Necrotic skeletal muscle damage ensued, nonetheless. In the skeletal muscles of the affected hindlimb, the micromorphological changes reported are in accordance with previously reported results from CLI patients and animal studies^42,43^. Because of the sustained long-term ischemia that could be achieved in the current work, the severity of skeletal muscle necrosis reported here is unprecedented.

Recent developments in the overall discipline of regenerative medicine offers new hope to the fight against CLI^44^. The emergence of several vasculogenic solutions including but not limited to vasculogenic, neurogenic and myogenic tissue reprogramming sets the stage for translational studies to test their efficacy in managing CLI^11,44–46^. Testing the efficacy of specific interventions in rescuing from necrotic ischemic myopathy associated with CLI depends on the availability of a robust large animal model of hind limb ischemia that involves necrotic muscle damage and allows long-term studies with minimal side effects.

## Conclusions

This work offers such a translational model, results from which are expected to be of value in traversing the regulatory path to testing safety and efficacy of the corresponding intervention in humans. In summary, this preclinical model more closely recapitulates the features characteristic of human CLI in a way that has been objectively validated by both non-invasive as well as invasive techniques. Thus, the reported CLI model lends itself to detailed mechanistic studies and interventional testing.

## Materials and methods

### Animals

All porcine experiments were performed in compliance with the protocols approved by the Indiana University School of Medicine Institutional Animal Care and Use Committee (SoM-IACUC) and The Ohio State University Institutional Laboratory Animal Care and Use Committee (ILACUC) under protocols 18085 and 2015A00000075, respectively. The study is reported in accordance with ARRIVE 2.0 guidelines. All the animals were included in the final data analysis. All methods were performed in accordance with the relevant institutional guidelines and regulations.

### Surgical procedure

#### Preoperative

Five 3-months old Yorkshire female pigs (60–70 lbs) were used. Animals were purchased from The Ohio State University farms and all health and vaccination certificates were provided and kept with the animal records. Every effort was made to minimize animal pain and distress as per the protocol and guidelines. Female pigs were used for several reasons: the prevalence of PAD is similar between men and women, but women more often have asymptomatic disease or atypical symptoms as well as severe ischemia^47,48^. Additionally, translational testing of therapeutics often neglects thorough investigation of the female sex^49^. PAD is more prevalent as age increases, and women make up a larger proportion of the elderly than men^50^. For all these reasons, we started with females. Given the small sample size, we chose to keep the sex homogenous. As it relates to the surgical approach reported in our work, there is no reason to suspect that it would not be equally applicable to males. Survival surgery was performed using standard sterile/aseptic techniques in accordance with IU IACUC and OSU ILACUC guidelines. Heart rate, respiration and body temperature were monitored while under anesthesia. Vital signs were recorded on an anesthesia and/or recovery log and placed in the animal’s medical record. Telazol® was administered intramuscularly for induction, then the pig was intubated and supported with mechanical ventilation. Anesthesia was maintained with isoflurane. Electrocardiogram (ECG), pulse, temperature, and blood pressure were monitored during the procedure. Transdermal Fentanyl patch was placed at the time of surgery and an intramuscular injection of buprenorphine was given prior to recovery per assessment of the attending veterinarian. All animals were evaluated for physical or vital sign abnormalities. Operative. Following positioning and sterile prep of the surgical site, a 2–3 inches infra inguinal skin incision was made overlying the femoral vessel after identifying the vessel location. Then the subcutaneous fat and fascia were dissected away to expose the artery. Once the femoral artery (FA) which equals to common femoral artery (CFA) in human is localized, two silk ligatures were placed, one proximal to the branching site and one at one inch proximal to the first ligature (Fig. 1). Intraoperative confirmation of the precise site of ligatures was done with arteriography and Doppler ultrasound. One inch of CFA was excised to ensure reflow was not possible. Hemostasis was achieved. At the conclusion, closure of the wound in layers was done using absorbable sutures. Skin closure was achieved using (3–0) non absorbable nylon suturing. Post-operative bupivacaine was injected into the incision site to minimize post-operative pain. Validation of ischemia. Hindlimb perfusion was assessed intraoperatively by arteriography to confirm the exact location of ligation and cessation of blood flow to the targeted hindlimb. Post-operative monitoring: During immediate post-operative period, pigs were observed continuously until extubated and sternally recumbent. Animals were monitored for signs of lethargy, inappetence, anorexia, and difficulty walking or movement of hind limbs to determine the need for additional analgesia and care. Once recovered, monitoring consisted of normal daily observations. There were no surgery related complications or life-threatening adverse effects reported. Imaging was performed at baseline pre and post transection of CFA, then once weekly up to d28 post ligation-Excision of CFA. At the end of the study, pigs were humanely euthanized as per the AVMA approved guidelines for euthanasia.

### Arteriography

Following positioning and sterile prep of the surgical site, a 2–4 cm incision was made in the ventral neck region and an arteriotomy performed on a carotid artery. A steerable arterial sheath (8.5 Fr) was introduced and advanced over a 0.035 inch guide wire through the descending aorta to the Aortic-iliac bifurcation. Catheter based arteriography was performed through the common carotid artery under fluoroscopy to deliver a catheter over the aortic bifurcation to the point where the CFA on the injured leg is ligated. Digital angiography of the peripheral vasculature was obtained which identified the femoral vessel and its branches supplying the hind limb pre and post CFA transection. We used Iohexol (350 mg Iodine/ml, Omnipaque, GE Healthcare) as a contrast material, and serial images obtained with fluoroscopy of both ischemic and contralateral hindlimbs.

### CT angiography

Under anesthesia, an IV catheter was placed prior to transporting the pig to CT imaging facility. In vivo CTA was performed at baseline, post-occlusion, and at week four post-occlusion using a 64-slice CT scanner with iodinated intravenous contrast agent Iohexol (350 mg iodine/ml, Omnipaque, GE Healthcare). Images were acquired at a slice thickness of 0.625 mm, at 300 mA and 120 kVp. Images were processed and 3D reconstructed for visualization by using Osirix DICOM viewer (Pixmeo SARL, Switzerland) software.

### Laser speckle imaging (LSI)

Perfusion imaging was performed using LSI system (Perimed Inc., Sweden)^51^. Color coded perfusion maps were captured from real-time recordings at different time points and average perfusion was calculated using PimSoft v1.4 software (Perimed Inc., Sweden). The ischemic tissues were identified as blue while normal perfusion ranged from yellow to red colors. Region of interests (ROI) from the collected LSI images were chosen. From the real-time graphs obtained, time-of-interest (TOI) was chosen to exclude motion related artifacts. Mean and standard deviation of perfusion data were obtained from the selected TOI perfusion data. Imaging was done for both ischemic and contralateral limbs.

### High resolution ultrasound and elastography imaging

Scanning axial view of both ischemic and non-ischemic hindlimbs was recorded using a linear array probe with a frequency range of 3–18 MHz (Noblus, Hitachi-Aloka Medical Corporation, Japan)^52,53^. Dual B-mode (gray scale images) and corresponding elastography mode (colored images) were acquired. Tissue elastography imaging allows the qualitative, non-invasive mapping of tissue stiffness^54^. Consistent and intermittent pressure with the ultrasound (US) probe on the scanned areas was maintained by the operator, that was controlled by indicator displayed in the US monitor, ranging from blue (stiff tissues) to red (soft tissues). Images from different anatomical areas in the thigh region with color information were selected for elasticity quantification. Measurements were analyzed from the extracted elastography images via colored pixel separation using ImageJ software (NIH) with reference to the scale in the images. The increase in muscle stiffness is represented by an increase in blue colored regions over time until d28 (sacrifice day).

### Color Doppler flow imaging

The tissue Doppler color flow imaging (CFI) feature of the ultrasound machine was used to obtain video clips with color-coded images representing blood flow^52^. This technique detects arteries and measures the flow velocity up to a depth of 10-15 cm. Real-time velocity profiles were used to measure systole (taller profile peaks) and diastole (shorter profile peaks) values^52^. Doppler assessment of the femoral artery and its branches in both ischemic and non-ischemic hind limb was done. Pulse velocity assesses arterial wall stiffness, and it is also important to determine local hemodynamic performance. Calculation of Doppler parameters (systolic/diastolic ratio, resistive index—RI and pulsatility index—PI) were done from the recorded CFI using standard equations^55^. The PI and RI were calculated from the Doppler ultrasound waves and they were derived from the maximum, minimum and mean doppler frequency shifts during a cardiac cycle as reported^56,57^:

$${\text{PI}} = ({\text{PSV}} - {\text{MDV}})/{\text{MV}}$$

and

$${\text{RI}} = {\text{ (PSV}} - {\text{MDV)}}/{\text{PSV}}$$

where *PSV* peak systolic velocity, *MDV* minimum diastolic velocity, *MV* mean velocity.

### Histology and immunohistochemistry

Skeletal muscle biopsies were collected from semimembranosus, vastus lateralis and gastrocnemius muscles on day 28 post occlusion in formalin. A systematic approach was followed to collect biopsies from 3 different parts (proximal, middle, and distal) of each muscle to show heterogeneity. Paraffin embedded samples were processed as previously described^58^. Briefly, the tissue sections (8 µm thick) were stained with hematoxylin for 5 min, washed in PBS and then counterstained with eosin for 2 min. The sections were dehydrated with graded alcohol and washed with xylene followed by mounting. For immunostaining, sodium citrate (pH 6.0) antigen retrieval, blocking with 10% normal goat serum at room temperature was performed, then incubated with rat laminin ab (MA1-06100; 1:200; ThermoFisher), rabbit vWF (PA5-16634; 1:200; ThermoFisher), rabbit NCAM1 ab (ab75813; 1:1000; abcam) and mouse MYH1 ab (67299-1-IG; 1:1000; ThermoFisher) overnight at 4 °C. Signal was visualized by subsequent fluorescence-tagged secondary antibodies (Alexa Fluor 488-tagged α-rat, 1:200, Alexa Fluor 568-tagged α-rabbit, 1:200, and Alexa Fluor 488-tagged α-mouse, 1:200) respectively with DAPI counterstained. Images were collected using Axioscanner Z1 (Zeiss).

### Microscopy and histology quantification

Imaging was done using axioscanner Z1 and the processing of the images was performed using an algorithm and analysis feature in Zen software (3.4). Multiple ROIs were randomly selected from each section of the images. The samples were taken from semimembranosus, vastus lateralis (thigh region) and gastrocnemius muscles (leg region). For H&E, 7 ROIs were selected from each sample. For immunofluorescence samples 10 ROIs were selected from each sample. Semi-automated quantification was done from H&E-stained images by segmentation feature in the Zen software. Longitudinal or oblique muscle fibers and neurovascular bundles were excluded during quantification by adjusting the settings in the software to above 100 μm^2^. The measurements included total fiber number, fiber size and minimal Feret’s diameter and morphological characteristics such as roundness^59^. Quantification of blood vessels number and NCAM1^+^ muscle fibers was performed in Zen software by counting the vessels or NCAM1^+^ fibers in each ROI and then taking the average to represent the entire sample.

### Statistical analyses

GraphPad Prism (GraphPad v9.0) software was used for statistical analyses. No statistical methods were used to predetermine sample size. Statistical analysis between two groups were performed using unpaired Student’s two-tailed *t* tests. *P* < 0.05 was considered statistically significant. Significance levels and *P* values were indicated in all relevant figures. Data were assumed to be normally distributed for all analyses conducted. Data for independent experiments are presented as mean ± SD unless otherwise stated.

### Ethical approval

All animal experiments were performed in compliance with the protocols approved by the Indiana University School of Medicine Institutional Animal Care and Use Committee (SoM-IACUC) and The Ohio State University Institutional Laboratory Animal Care and Use Committee (ILACUC) under protocols 18085 and 2015A00000075, respectively.

## Supplementary Information

Supplementary Information.

## Data Availability

The datasets generated and/or analyzed during the current study are available from the corresponding authors on reasonable request.

## Abbreviations

CLI: Critical limb ischemia
PAD: Peripheral arterial diseases
CFA: Common femoral artery
SFA: Superficial femoral artery
LSI: Laser speckle imaging
CTA: Computed tomography angiography
US: Ultrasound
CFI: Color flow imaging
PI: Pulsatility index
RI: Resistive index
