# Supplementary material for: Robust critical limb ischemia porcine model involving skeletal muscle necrosis
Source: Sci Rep. 2023 Jul 18;13:11574. doi: 10.1038/s41598-023-37724-7 (PMC10354195; doi:10.1038/s41598-023-37724-7)
Supplement: Supplementary file 1 — Supplementary Information. [file 41598_2023_37724_MOESM1_ESM.pdf]

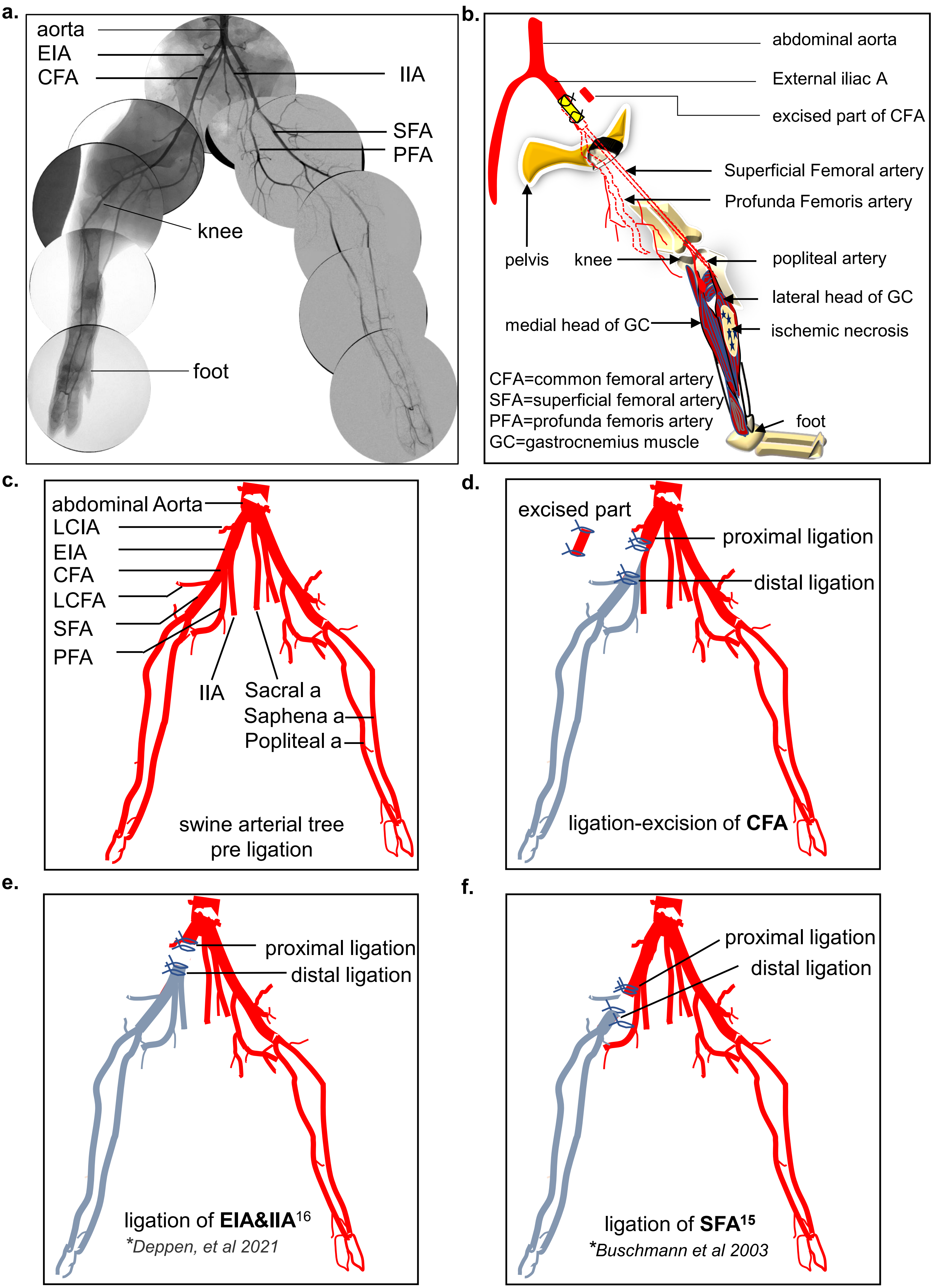
a.
aorta
EIA
CFA
IIA
SFA
PFA
knee
foot
b.
abdominal aorta
External iliac A
excised part of CFA
Superficial Femoral artery
Profunda Femoris artery
pelvis
knee
popliteal artery
lateral head of GC
medial head of GC
ischemic necrosis
CFA=common femoral artery
SFA=superficial femoral artery
PFA=profunda femoris artery
GC=gastrocnemius muscle
foot
c.
abdominal Aorta
LCIA
EIA
CFA
LCFA
SFA
PFA
IIA
Sacral a
Saphena a
Popliteal a
swine arterial tree
pre ligation
d.
excised part
proximal ligation
distal ligation
ligation-excision of **CFA**
e.
proximal ligation
distal ligation
ligation of **EIA&IIA**[16]
Deppen, et al 2021
f.
proximal ligation
distal ligation
ligation of **SFA**[15]
Buschmann et al 2003

Figure S1

Fig S1: Serial reconstructed arteriography of the swine hindlimb. **a.** Reconstructed serial fluoroscopy images of both hind limbs to depict the arterial tree starting from abdominal aorta to the smaller vessels of the foot with its corresponding anatomical regions. **b.** Schematic diagram of swine hind limb vascular tree depicting the 1″ CFA removed by ligation- excision. **c.** Swine arterial tree. **d**. ligation–excision of CFA as performed in this work. **e-f.** Different models of published hindlimb ischemia as reference, **e.** Ligation of the external and internal iliac arteries. **f.** Ligation of the SFA only. LCIA=lateral circumflex iliac artery; EIA=external iliac artery; IIA=internal iliac artery; CFA=common femoral artery; LCFA=lateral circumflex femoral Artery; SFA=superficial femoral artery; PFA=profunda femoris artery; GC=gastrocnemius muscle.

Figure S1

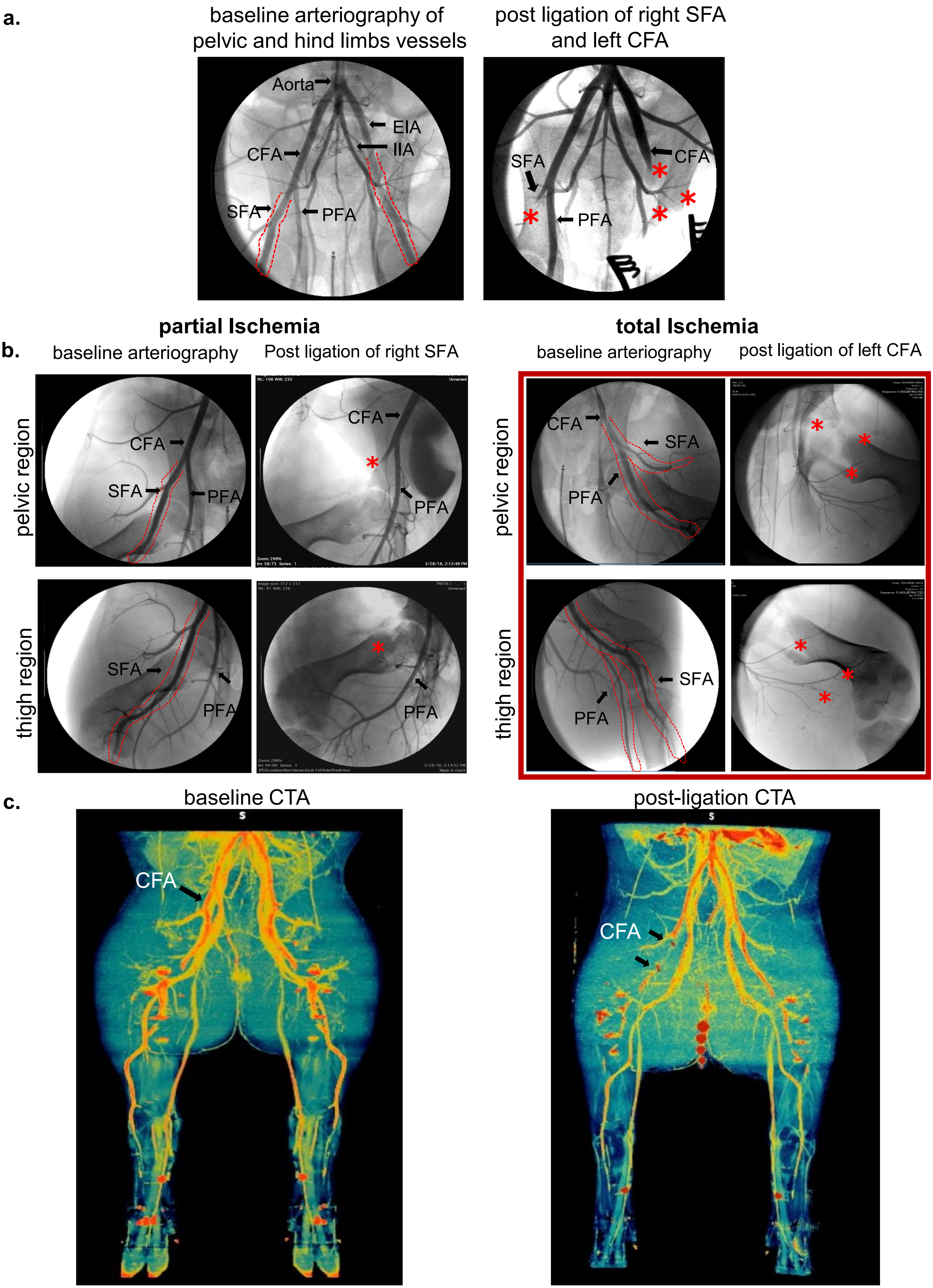
a.
baseline arteriography of pelvic and hind limbs vessels
post ligation of right SFA and left CFA
Aorta
EIA
IIA
CFA
SFA
PFA
b.
partial Ischemia
total Ischemia
baseline arteriography
Post ligation of right SFA
post ligation of left CFA
pelvic region
thigh region
c.
baseline CTA
post-ligation CTA
s

Figure S2

Fig S2: X-ray arteriographic characterization of hindlimb ischemia (partial vs total ischemia). **a.** Representative arteriography images showing baseline pig hindlimb arterial tree on the left. Right image is demonstrating the different levels of ischemia induction (partial ischemia by SFA transection compared t complete ischemia achieved by CFA transection). The asterisks are marking the affected arteries. EIA=external iliac artery; IIA=internal iliac artery; CFA=common femoral artery; SFA=superficial femoral artery; PFA=profunda femoris artery. **b.** Arteriography images of the arterial tree at different anatomical region (pelvis and thigh) demonstrating the different level of ischemia. Left side panel showing partial ischemia by transection of SFA with normal flow in the PFA. Right side panel showing complete absence of flow in both SFA and PFA that was noticed directly at d0 early post-transection demonstrating the successful induction of ischemia achieved by transection of CFA. The red dotted lines are marking the targeted arteries. The asterisks are marking the affected arteries. **c.** Representative CT angiography images illustrating baseline pig hindlimb arterial tree (left) and the site of excised part of the CFA (right, marked by double arrow).

Figure S2

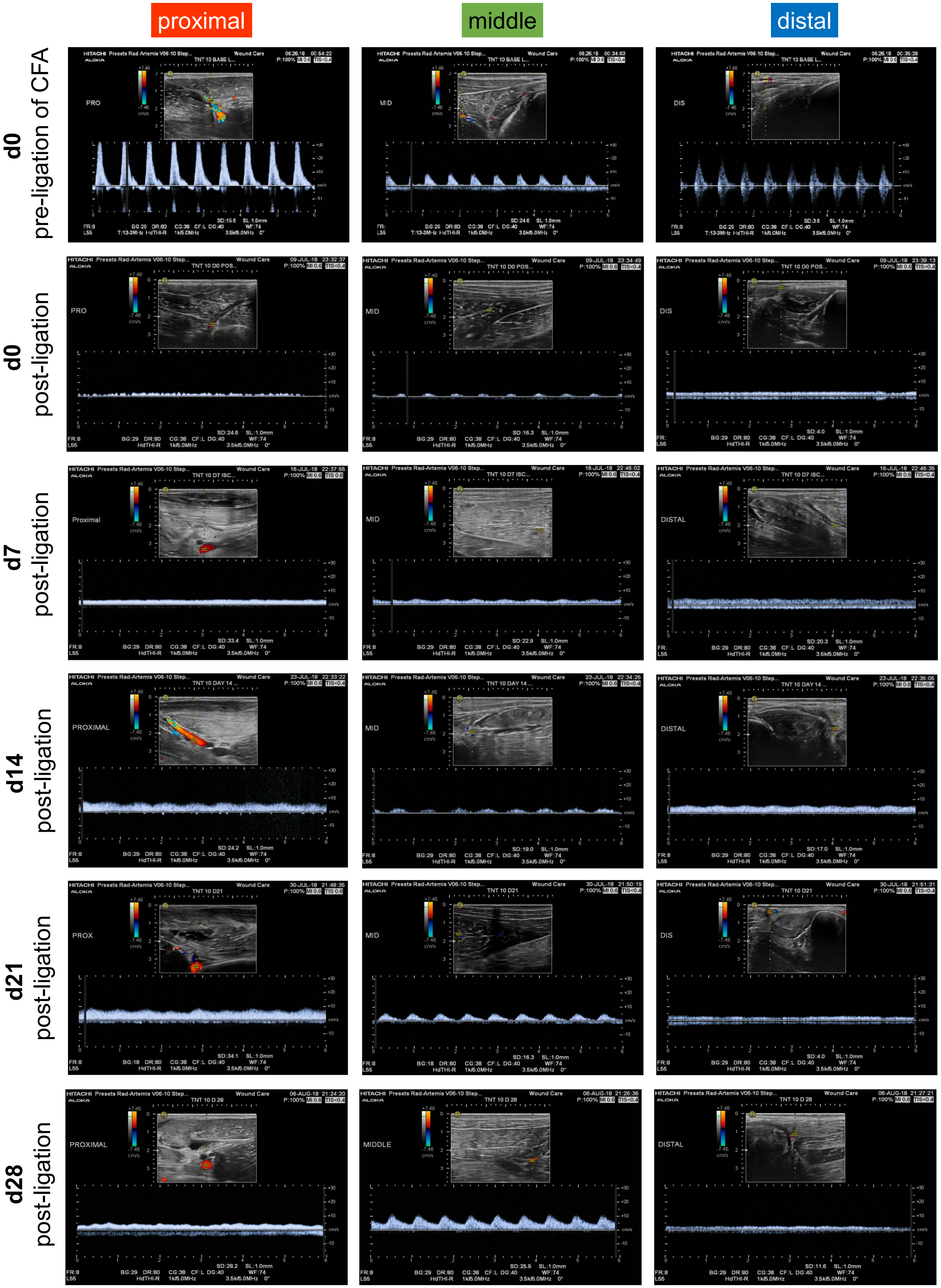

proximal
middle
distal
d0 pre-ligation of CFA
d0 post-ligation
d7 post-ligation
d14 post-ligation
d21 post-ligation
d28 post-ligation

Figure S3

Fig S3: Sustained hindlimb ischemia during the first four weeks after ligation-excision of CFA. Doppler images (still frame from video record) demonstrating normal Doppler waves at weekly recording started at day 0 pre-ligation of the CFA until day 28 pos-ligation. Following ligation-excision of the CFA, the waves are eliminated validating ischemia. CFA=common femoral artery.

Figure S3

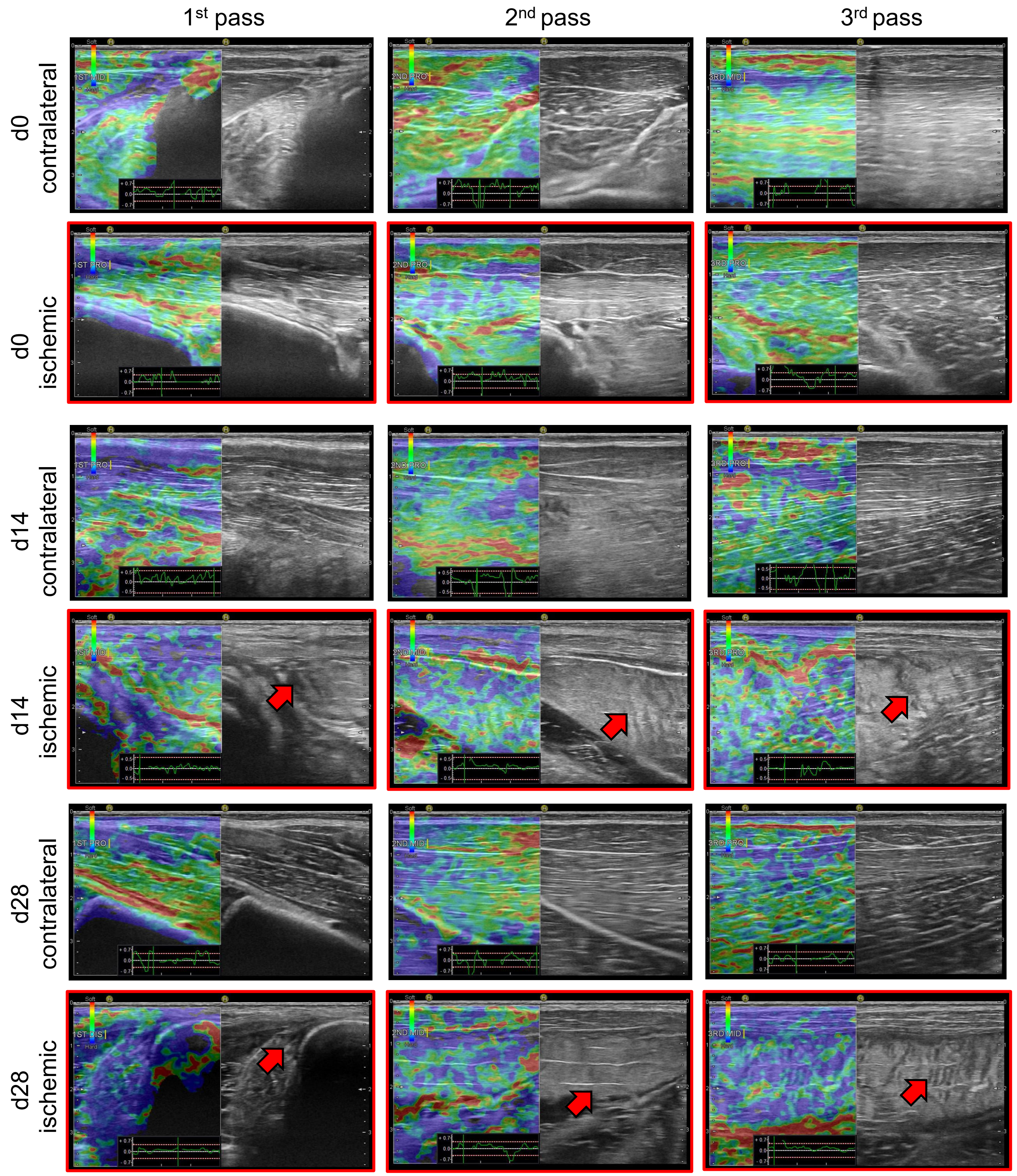
1st pass
2nd pass
3rd pass
d0 contralateral
d0 ischemic
d14 contralateral
d14 ischemic
d28 contralateral
d28 ischemic

Figure S4

Fig S4: Structural (B-mode images, grey scale ultrasound) and biomechanical (elastography, color ultrasound) of the hindlimb skeletal muscles in response to experimental ischemia. Co-registered (B-mode and elastography) ultrasound images characterizing baseline homogenous distribution pattern of skeletal muscle echo intensity (B-mode) and biomechanical strain (elastography) at d0 before induction of ischemia in paired ischemic and contralateral hindlimbs. Post-surgical weekly assessment illustrating the progression of ischemic myopathic changes in the form of increased muscle echo intensity (red arrows). The increase in muscle stiffness is represented by increase in blue colored regions over time until d28 (sacrifice day).

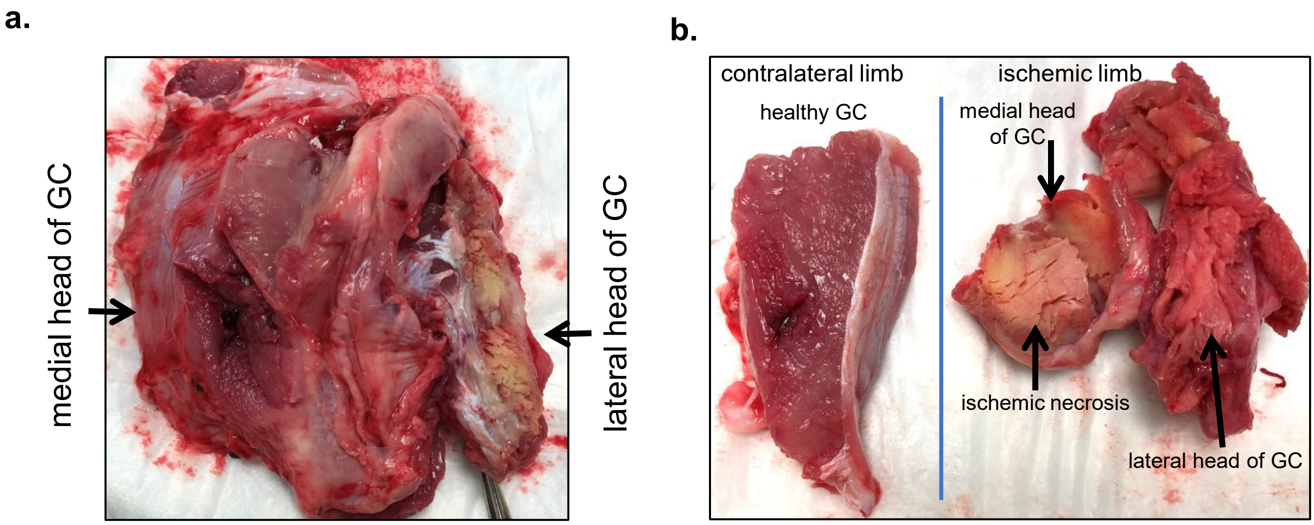

Fig S5: Macroscopic validation of ischemic muscle necrosis. **a.** Digital images (d28 post-ischemia) showing the proof of muscle ischemic necrosis in both heads of gastrocnemius muscle of the ischemic limb with more necrosis in the lateral head in (pale color and hard texture characteristic of fibrosis) **b.** Digital images (d28 post-ischemia) showing the contralateral health gastrocnemius muscle (viable and healthy, red color with soft fleshy texture) compared to the ischemic gastrocnemius in the ischemic limb (pale color and hard texture characteristic of fibrosis). GC=gastrocnemius muscle.

Figure S5

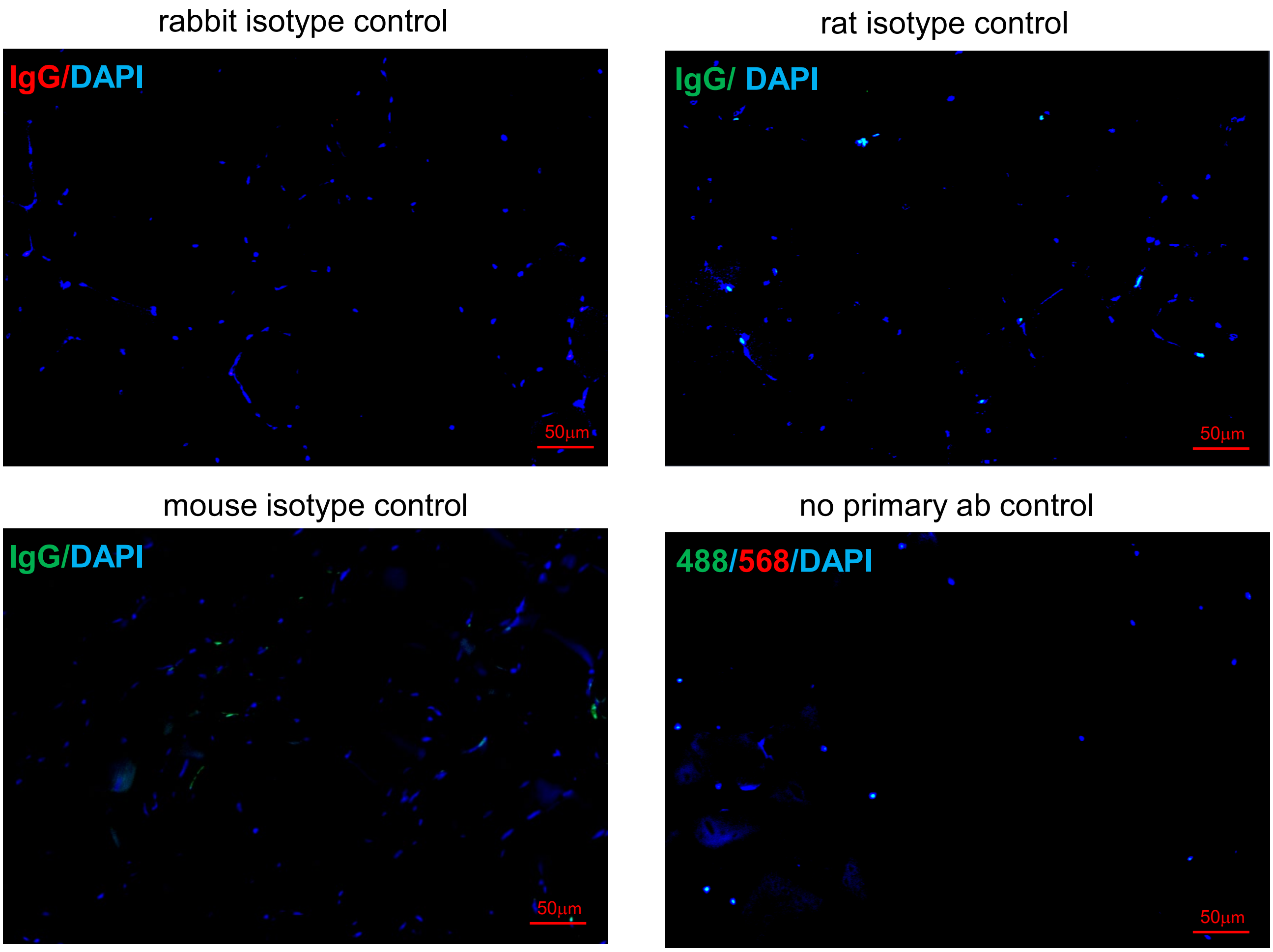

Fig S6: Isotype controls for all antibodies used for immunofluorescence staining .

Figure S6
